# Lifestyle Intervention Enabled by Mobile Technology on Weight Loss in Patients With Nonalcoholic Fatty Liver Disease: Randomized Controlled Trial

**DOI:** 10.2196/14802

**Published:** 2020-04-13

**Authors:** Su Lin Lim, Jolyn Johal, Kai Wen Ong, Chad Yixian Han, Yiong Huak Chan, Yin Mei Lee, Wai Mun Loo

**Affiliations:** 1 Dietetics Department National University Hospital National University Health System Singapore Singapore; 2 Biostatistics Unit Yong Loo Lin School of Medicine National University of Singapore Singapore Singapore; 3 Department of Medicine, Gastroenterology and Hepatology National University Hospital National University Health System Singapore Singapore

**Keywords:** diet, NAFLD, mHealth, mobile app, weight loss, liver enzymes, lifestyle intervention

## Abstract

**Background:**

The prevalence of nonalcoholic fatty liver disease (NAFLD) reaches up to 30% in the Asian adult population, with a higher prevalence in obese patients. Weight reduction is typically recommended for patients at high risk or diagnosed with NAFLD, but is a challenge to achieve.

**Objective:**

We aimed to evaluate the effect of a lifestyle intervention with a mobile app on weight loss in NAFLD patients.

**Methods:**

This prospective randomized controlled trial included 108 adults with NAFLD confirmed by steatosis on ultrasound and a body mass index ≥23 kg/m^2^ who were recruited from a fatty liver outpatient clinic. The patients were randomly allocated to either a control group (n=53) receiving standard care, consisting of dietary and lifestyle advice by a trained nurse, or an intervention group (n=55) utilizing the Nutritionist Buddy (nBuddy) mobile app in addition to receiving dietary and lifestyle advice by a dietitian. Body weight, alanine aminotransferase (ALT), aspartate aminotransferase (AST), waist circumference, and blood pressure were measured at baseline, and then at 3 and 6 months. Intention-to-treat and per-protocol analyses were used for statistical comparisons.

**Results:**

The intervention group had a 5-fold higher likelihood (relative risk 5.2, *P*=.003, 95% CI 1.8-15.4) of achieving ≥5% weight loss compared to the control group at 6 months. The intervention group also showed greater reductions in weight (mean 3.2, SD 4.1 kg vs mean 0.5, SD 2.9 kg; *P*<.001), waist circumference (mean 2.9, SD 5.0 cm vs mean –0.7, SD 4.4 cm; *P*<.001), systolic blood pressure (mean 12.4, SD 14.8 mmHg vs mean 2.4, SD 12.4 mmHg; *P*=.003), diastolic blood pressure (mean 6.8, SD 8.9 mmHg vs mean –0.9, SD 10.0 mmHg; *P*=.001), ALT (mean 33.5, SD 40.4 IU/L vs mean 11.5, SD 35.2 IU/L; *P*=.004), and AST (mean 17.4, SD 27.5 U/L vs mean 7.4, SD 17.6 IU/L, *P*=.03) at 6 months.

**Conclusions:**

Lifestyle intervention enabled by a mobile app can be effective in improving anthropometric indices and liver enzymes in patients with NAFLD. This treatment modality has the potential to be extended to a larger population scale.

**Trial Registration:**

Australian New Zealand Clinical Trials Registry ACTRN12617001001381; https://tinyurl.com/w9xnfmp

## Introduction

Nonalcoholic fatty liver disease (NAFLD) is characterized by excessive accumulation of fat in the liver that is not directly caused by alcohol consumption. NAFLD represents a clinicopathological spectrum that consists of hepatic steatosis and nonalcoholic steatohepatitis (NASH), and varying in degrees of inflammation and fibrosis [1,2]. Up to 20% of patients with NASH progress to cirrhosis and develop end-stage liver disease and associated complications [3,4].

With the epidemic surge in obesity and type 2 diabetes, the prevalence of NAFLD is on the rise, which is increasingly being recognized as a major cause of morbidity and mortality [5]. From 2002 to 2012, the number of patients undergoing liver transplantation for NASH-related hepatocellular carcinoma increased by 4-fold [6]. NASH is now poised to become the leading cause of hepatocellular carcinoma and indication for liver transplantation in the United States [6].

In Asia, an estimated 20%-30% of the adult population have been diagnosed with NAFLD, with a higher prevalence among patients with obesity [7]. The increase in prevalence can be attributed to a shift in dietary and lifestyle habits due to rapid globalization [8]. Notably, Asians are more susceptible to NAFLD at equivalent levels of overnutrition as compared to their Western counterparts, in part due to differences in the adiposity-muscle composition [1]. In view of the increased risk of morbidity in the Asian population, the World Health Organization (WHO) recommends 23 kg/m^2^ as the body mass index (BMI) cut-off point for clinical action [9], while the International Diabetes Federation recommends waist circumference cut-off points of 90 cm and 80 cm for Asian men and women, respectively [10].

Despite pharmacological advances, lifestyle interventions remain a fundamental approach for the therapeutic management of NAFLD [11-13]. Suzuki et al [14] demonstrated the ability of a 5% weight loss in alleviating high serum alanine aminotransferase (ALT) and reducing hepatic steatosis in patients with NAFLD. However, the success of such weight loss interventions is dependent on the intensity of nutrition counseling and frequency of visits to dietitians and exercise therapists [15]. This renders the treatment modality resource-intensive and costly with high attrition rates, thereby limiting reach and scalability [16]. Mobile apps have recently gained increasing popularity in facilitating weight loss [17,18]. Self-monitoring of dietary intake and physical activity achieved through the convenience of mobile devices has been associated with greater reductions in energy intake and weight loss [19,20]. By mitigating the barriers associated with committing to repeated in-house nutrition and exercise therapy sessions, mobile apps increase the potential of expanding the reach and efficacy of lifestyle interventions.

To our knowledge, there is a paucity of research investigating the effects of integrating mobile apps into specialized weight loss programs for patients with NAFLD, with no full-sized randomized controlled trials published to date. Furthermore, a systematic review identified that none of the commercially available weight management mobile apps available included all major aspects of evidence-based strategies of self-monitoring, goal-setting, motivational strategies, healthy eating and physical activity support, social support, health or weight assessment, and personalized feedback, along with the involvement of health care professionals and formal scientific evaluation [21]. Accordingly, the objectives of the present study were to evaluate the effect of a lifestyle intervention consisting of diet and physical activity enabled by a mobile app that integrates a spectrum of evidence-based strategies along with health care professional involvement in facilitating weight loss and improving relevant health indicators in patients with NAFLD.

## Methods

### Study Participants

This parallel randomized controlled trial was conducted between July 2017 and November 2018 at National University Hospital (NUH), a tertiary university hospital in Singapore. Patients were recruited from an NAFLD clinic in NUH through referrals from clinicians after screening. NAFLD was defined as the presence of hepatic steatosis, either determined by imaging or histology, in the absence of secondary causes of hepatic fat accumulation such as significant alcohol consumption, long-term use of a steatogenic medication, or monogenic hereditary disorders, in accordance with the American Association for the Study of Liver Diseases guidelines [11]. Adults above 21 years of age who were diagnosed with NAFLD after excluding secondary causes of liver fat accumulation, and with a BMI≥23 kg/m^2^, able to read and write in English, and who owned a smartphone with a data plan were included in the study. Exclusion criteria were consumption of more than one and a half times the limit of alcohol recommended for the population (15 g/day for women and 30 g/day for men) and those with infections of hepatitis B or C virus. Patients who were pregnant, receiving hepatotoxic medication, with cirrhosis, poorly controlled diabetes mellitus (HbA1c>10%), diabetes requiring insulin, cardiovascular event in the past 6 months, stage 4 and above kidney disease, concomitant liver disease, depression, untreated hypothyroidism, heart failure, and clinically or biochemically recognized systemic diseases were also excluded from the study. Written informed consent was obtained from each patient before enrolment. All participants were provided with a standardized model digital weighing scale (Omron HN-286, Japan) for self-monitoring of weight. This study was conducted in accordance with the Declaration of Helsinki, and received ethical approval from the National Healthcare Group Domain Specific Review Board in Singapore. The trial was registered at the Australian New Zealand Clinical Trials Registry (ACTRN12617001001381).

### Randomization and Blinding

Stratified randomization of screened participants was carried out by gender, age (<40 years or ≥40 years), and BMI (<27.5 kg/m^2^ or ≥27.5 kg/m^2^). Within each stratum, participants were assigned to either the control or intervention group based on drawing from sealed opaque envelopes with an allocation ratio of 1:1, prepared by a third party not involved in the study and blinded to the study objectives according to the Consolidated Standards of Reporting Trials (CONSORT) statement (see Multimedia Appendix 1) [22].

### Mobile App Group (Intervention)

**Figure 1:**
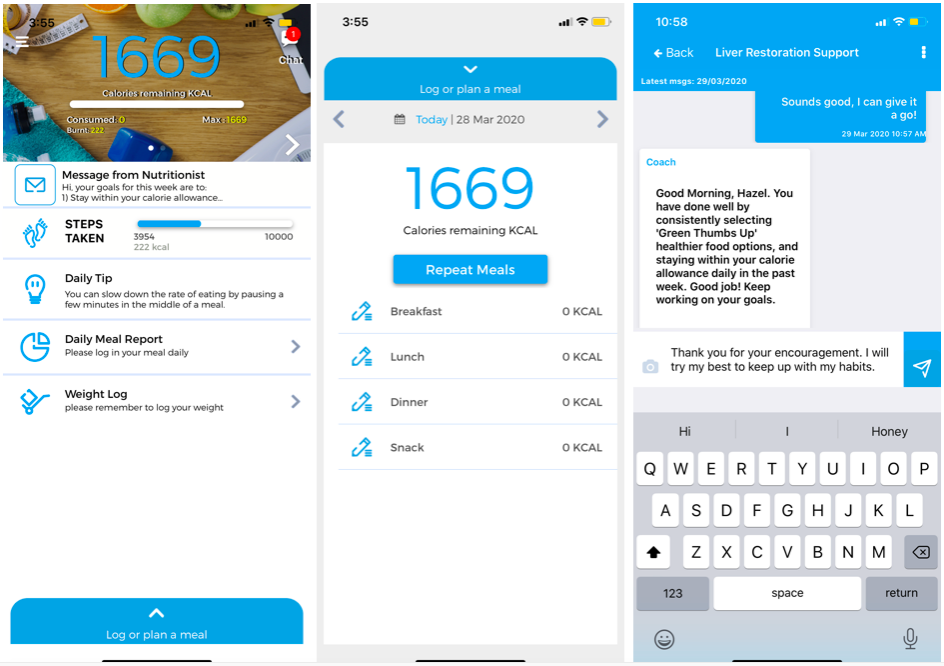
Screenshot of nBuddy homepage, food log page, and dietitian support chat channel from left to right, respectively.

To evaluate the effect of lifestyle intervention enabled by a mobile app in facilitating weight loss, participants allocated to the intervention group were each provided with advice on dietary and physical activity modification by a dietitian via a single face-to-face session in the clinic followed by remote support through a mobile app for a 6-month period. They were taught how to utilize the Nutritionist Buddy (nBuddy) mobile app to track their diet and physical activity and induce behavioral changes to achieve optimal weight (Figure 1). The app was conceptualized by the principal investigator (SL) and developed by Verita Analytics in Singapore. nBuddy was developed using the Obesity-Related Behavioral Intervention Trials model for behavioral treatment as a framework for translating behavioral science discoveries into treatments, which is a flexible and robust process to design, conduct, and evaluate mobile app–based behavioral interventions [23]. The app is available commercially in app stores, with basic features accessible for free. Payment is required to unlock additional features such as videos, daily tips, and nutritionist support. Full features of the app were made available to the intervention participants as part of collaboration with Verita Analytics. The following set of in-built functions in nBuddy represent an amalgamation of evidence-based behavioral modification strategies to promote weight loss or maintenance.

The app includes a food diary logging system, coupled with individualized caloric goals based on the user’s age, gender, and physical activity level, allowing for self-monitoring of intake [24]. Automatic recording of daily steps is achieved via syncing with the built-in pedometer of users’ mobile devices to enable self-monitoring of physical activity [25]. The step goal increases automatically each week, from an initial goal of 3000 steps to 10,000 steps by the third week of usage. A range of physical activities can be logged manually if exercises were done in the absence of mobile devices. A weight logging function encourages the self-tracking of weight loss progression [26]. A dashboard enables dietitians to monitor users’ input (ie, food intake and physical activity) and progress (ie, weight) to provide real-time feedback and encouragement [27]. A peer support chat channel allows users to connect with selected family members and peers to bolster user motivation [25,27]. A video viewing function delivers weekly educational clips [28]. An automated response system evaluates the suitability of food choices and provides instantaneous feedback, generating a list of healthier and culturally appropriate alternatives via an algorithm [29]. Daily, weekly, and monthly graph reports on weight, calorie intake, and steps facilitate the tracking of progress [24]. Finally, scripted daily tips and timed automated reminders prompt users to log in daily meal intake and twice weekly weight [30].

### Standard Care Group (Control)

Participants randomized to the control group were provided with usual standard care, which consisted of advice on dietary and physical activity modification as per American Heart Association guidelines [31] by a nurse trained in diet counseling via a single face-to-face session in the NAFLD clinic. The syllabus was similar to that provided in the face-to-face visit with the dietitian in the intervention group. This was part of the routine clinical service offered to overweight or obese NAFLD patients seen at the NAFLD clinic.

### Outcome Measurements

All outcomes were part of routine measurements taken by trained nurses and blood tests conducted at the outpatient NAFLD clinic. Assessors were not blinded to the groups allocated to the study participants. Body weight was measured using a calibrated digital weighing machine (Seca 767, Germany) to the nearest 0.1 kg. Height was measured in meters to two decimal points using the stadiometer attached to the Seca scale and the corresponding BMI was calculated. Waist circumference was measured using a tape measure at the midpoint between the lower margin of the last palpable rib and the top of the iliac crest as recommended by the WHO [32]. ALT and aspartate aminotransferase (AST) levels were determined by an automated kinetic method. Blood pressure (for participants with hypertension) was measured using an automatic blood pressure monitor (Carescape Dinamap V100; GE Healthcare, Chicago, IL, USA). Participants’ characteristics such as age, gender, ethnicity, and existing relevant comorbidities were also collected at baseline. App utilization data were collected through the app developer.

### Statistical Analysis

Studies that provided nutrition therapy interventions targeted at weight loss in patients with NAFLD were referenced for sample size calculation [14,24]. The primary unit of interest was weight loss of at least a 5% by 6 months. It was postulated that 10% of the control subjects will achieve this successful outcome and the intervention would increase this rate by 4-fold [33]. With 90% power at a 5% significance level, and allowing for a 10% dropout rate, a sample size of 100 (50 per group) would be required. Data analyses were performed using SPSS for Windows version 21.0 (SPSS Inc, Chicago, IL, USA). Results are expressed as mean and SD for normally distributed variables, and as the median and interquartile range for variables that did not satisfy normality criteria. Categorical data are expressed as frequencies and percentages. To compare baseline characteristics, and 3- and 6-month changes between groups, Chi square and independent samples *t* tests were used for categorical and continuous variables, respectively. Between-group differences in the numerical and binary outcomes were compared using a general linear model and Poisson regression model, respectively, adjusting for age, gender, and ethnicity. Between-group Cohen *d* effect sizes were calculated with the formula (M_intervention_–M_control_)/SD_pooled_, where M_intervention_ and M_control_ are the mean changes in outcomes from baseline in the intervention and control groups, respectively.

## Results

### Participants

**Figure 2:**
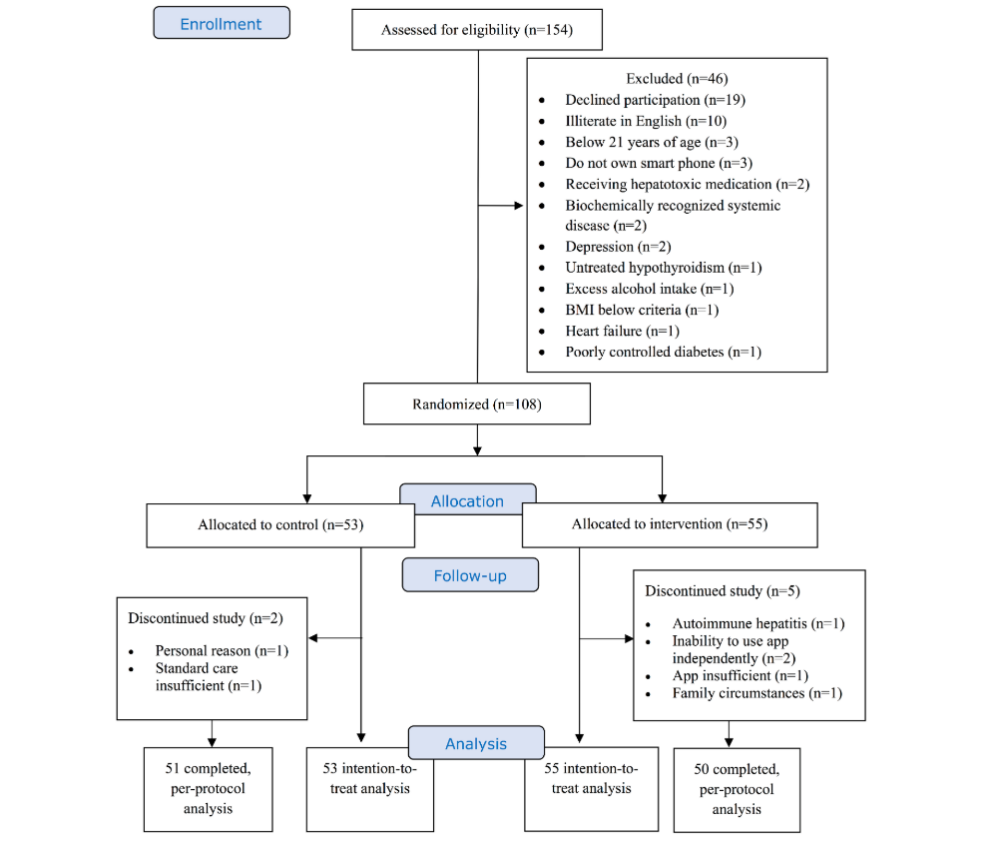
Consolidated Standards of Reporting Trials (CONSORT) study flow diagram. BMI: body mass index.

A total of 154 referred subjects were screened for participation. Forty-six did not meet the eligibility criteria, primarily due to refusal to participate and a lack of English literacy. The remaining 108 subjects were enrolled and randomized (55 to the intervention group and 53 to the control group). Seven of the enrolled participants withdrew from the study, including 5 (4.6%) from the intervention group and 2 (1.9%) from the control group. A total of 101 patients completed the study, with 50 allocated to the intervention group (Figure 2).

### Baseline Characteristics

The baseline characteristics of the participants are summarized in Table 1. There were no significant differences between groups in terms of baseline weight and other characteristics such as presence of relevant comorbidities, age, weight, and clinical and biochemical outcomes of interest. Per-protocol analysis was carried out on the 101 patients who completed the study, and intention-to-treat analysis was carried out among all 108 patients assigned to the original groups, where data were available for each of the parameters.

**Table 1:** Baseline characteristics of study participants.

Variable		Intention-to-treat				Per-protocol		
		Control (n=53)	Intervention (n=55)	*P* value^a^	Control (n=51)		Intervention (n=50)	*P* value^a^
**Gender, n (%)**				.30				.37
	Male	36 (68)	32 (58)		35 (69)		30 (60)	
	Female	17 (32)	23 (42)		16 (31)		20 (40)	
**Ethnicity, n (%)**				.21				.15
	Chinese	41 (77)	42 (76)		39 (77)		39 (78)	
	Malay	9 (17)	6 (11)		9 (18)		5 (10)	
	Indian	0 (0)	4 (7)		0 (0)		4 (8)	
	Others	3 (6)	3 (6)		3 (6)		2 (4)	
Diabetes, n (%)		19 (36)	11 (20)	.66	18 (35)		10 (20)	.09
Hyperlipidemia, n (%)		40 (76)	39 (71)	.59	40 (78)		37 (74)	.60
Hypertension, n (%)		37 (70)	38 (69)	.94	37 (73)		36 (72)	.95
Age (years), mean (SD)		46.2 (10.1)	46.8 (11.1)	.76	46.1 (10.3)		46.2 (11.0)	.96
Weight (kg)		86.1 (19.4)	81.5 (15.2)	.17	86.6 (19.6)		82.0 (15.7)	.20
BMI^b^ (kg/m^2^)		30.8 (4.8)	30.1 (4.0)	.04	30.9 (4.8)		30.1 (4.1)	.37
Waist circumference (cm), mean (SD)		101.7 (11.4)	98.6 (9.0)	.14	101.9 (11.6)		98.9 (10.2)	.17
ALT^c^ (IU/L), mean (SD)		78.1 (46.7)	75.6 (43.3)	.77	75.3 (40.9)		76.8 (41.8)	.85
AST^d^ (IU/L), mean (SD)		50.1 (28.8)	49.1 (28.4)	.86	48.6 (26.2)		48.6 (25.7)	>.99
Systolic blood pressure (mmHg), mean (SD)		135 (12)	134 (14)	.72	140 (10)		141 (10)	.58
Diastolic blood pressure (mmHg), mean (SD)		76 (9)	75 (11)	.46	78 (10)		79 (10)	.67

^a^Chi square or independent samples *t* test as appropriate.

^b^BMI: body mass index.

^c^ALT: alanine aminotransferase.

^d^AST: aspartate aminotransferase.

### Weight Loss

There was a significantly greater number of participants who achieved ≥5% weight loss in the intervention as compared to the control group at both 3 and 6 months (Table 2). After adjustment for age, gender, and ethnicity, the intention-to-treat and per-protocol analyses showed that use of the mobile**-**enabled lifestyle intervention program was independently associated with a higher likelihood of achieving a ≥5% weight loss at 3 and 6 months when compared with standard care.

**Table 2:** Proportion of patients with ≥5% weight loss in the control and intervention groups.

Analysis		Control^a^, n (%)	Intervention^b^, n (%)	Unadjusted RR^c^ (95% CI)	*P* value	Adjusted^d^ RR (95% CI)	*P* value
**Intention-to-treat**							
	3 months	4 (8)	14 (25)	3.4 (1.1-10.2)	.03	3.5 (1.1-10.9)	.03
	6 months	4 (8)	22 (40)	5.3 (1.8-15.3)	.002	5.2 (1.8-15.4)	.003
**Per-protocol**							
	3 months	4 (8)	14 (28)	3.6 (1.2-10.8)	.03	3.8 (1.3-11.6)	.02
	6 months	4 (8)	22 (44)	5.6 (1.9-16.3)	.002	5.8 (2.0-16.9)	.001

^a^N=53 for intention-to treat; N=51 for per-protocol.

^b^N=55 for intention-to-treat; N=50 for per-protocol.

^c^RR: relative risk; the control group is the reference (1.00).

^d^Adjusted for age, gender, ethnicity.

Table 3 and Table 4 show that participants in the intervention group lost significantly more weight than those in the control group at 3 and 6 months based on the intention-to-treat and per-protocol analysis, respectively. The significantly greater improvements in both absolute and percentage weight loss in the intervention group were more pronounced at 6 months as compared to 3 months, with large between-group effect sizes in percentage weight loss at both 3 and 6 months (Cohen *d*>0.8 for all). Reductions in BMI were also significantly greater in the intervention group at 3 and 6 months. Results of the univariate analyses remained significant (*P*<.001) after further adjustments for age, gender, and ethnicity in both analyses.

**Table 3:** Mean (SD) changes in anthropometric, biochemical, and clinical outcomes in patients with nonalcoholic fatty liver disease from baseline at 3 and 6 months after enrolment based on intention-to-treat analysis.

Variable		n	Change from baseline		Between-group differences			
			Control	Intervention	Mean difference (95% CI)	*P* value (unadjusted)	*P* value (adjusted^a^)	Cohen *d*
**Weight, kg**								
	3 months	108	–0.8 (2.1)	–3.2 (3.1)	2.3 (1.3-3.3)	<.001	<.001	0.91
	6 months	108	–0.5 (2.9)	–3.2 (4.1)	2.6 (1.3-4.0)	<.001	<.001	0.76
**Weight, %**								
	3 months	108	–0.9 (2.5)	–3.7 (3.7)	3.0 (1.8-4.2)	<.001	<.001	0.95
	6 months	108	–0.6 (3.5)	–4.4 (5.6)	3.8 (2.0-5.5)	<.001	<.001	0.81
**BMI^b^, kg/m^2^**								
	3 months	108	–0.4 (0.8)	–1.3 (1.1)	0.9 (0.5-1.2)	<.001	<.001	0.66
	6 months	108	–0.3 (1.1)	–1.3 (1.4)	1.0 (0.5-1.5)	<.001	<.001	0.79
**Waist circumference, cm**								
	3 months	105	0.4 (4.7)	–3.4 (5.1)	3.8 (1.9-5.7)	<.001	<.001	0.77
	6 months	105	0.7 (4.4)	–2.9 (5.0)	3.6 (1.8-5.5)	<.001	<.001	0.76
**ALT^c^, IU/L**								
	3 months	75	–20.7 (32.2)	–37.2 (37.6)	16.5 (0.4-32.7)	.045	.04	0.47
	6 months	103	–11.5 (35.2)	–33.5 (40.4)	22.0 (7.2-36.8)	.004	.006	0.58
**AST^d^, IU/L**								
	3 months	76	–11.1 (19.1)	–20.2 (26.9)	9.1 (–1.7-19.9)	.10	.07	0.39
	6 months	104	–7.4 (17.6)	–17.4 (27.5)	10.0 (1.0-19.0)	.03	.03	0.43
**Systolic blood pressure (mmHg)**								
	3 months	63	1.1 (12.6)	–13.7 (14.9)	14.8 (7.9-21.8)	<.001	<.001	1.07
	6 months	72	–2.4 (12.4)	–12.4 (14.8)	10.1 (3.6-16.5)	.003	.008	0.74
**Diastolic blood pressure (mmHg)**								
	3 months	63	2.3 (7.7)	–6.3 (9.8)	8.6 (4.1-13.1)	<.001	<.001	0.98
	6 months	72	0.9 (10.0)	–6.8 (8.9)	7.7 (3.2-12.1)	.001	.003	0.81

^a^Adjusted for age, gender, and ethnicity.

^b^BMI: body mass index.

^c^ALT: alanine aminotransferase.

^d^AST: aspartate aminotransferase.

**Table 4:** Mean (SD) changes in anthropometric, biochemical, and clinical outcomes in patients with nonalcoholic fatty liver disease from baseline at 3 and 6 months after enrolment based on per-protocol analysis.

Variable		n	Change from baseline			Between-group differences					
			Control	Intervention	Mean difference (95% CI)		*P* value (unadjusted)	*P* value (adjusted^a^)		Cohen *d*	
**Weight, kg**											
	3 months	101	–1.0 (2.0)	–3.2 (3.1)	2.3 (1.2-3.3)		<.001	<.001		0.88	
	6 months	101	–0.7 (2.8)	–4.1 (3.8)	3.5 (2.1-4.8)		<.001	<.001		1.05	
**Weight, %**											
	3 months	101	–1.1 (2.4)	–4.0 (3.7)	2.9 (1.7-4.1)		<.001	<.001		0.93	
	6 months	101	–0.7 (3.3)	–5.1 (4.5)	4.4 (2.9-6.0)		<.001	<.001		1.11	
**BMI^b^, kg/m^2^**											
	3 months	101	–0.4 (0.8)	–1.4 (1.1)	1.0 (0.6-1.3)		<.001	<.001		1.04	
	6 months	101	–0.3 (1.0)	–1.4 (1.5)	1.1 (0.6-1.6)		<.001	<.001		0.86	
**Waist circumference, cm**											
	3 months	99	0.24 (4.69)	–3.57 (5.14)	3.8 (1.8-5.8)		<.001	<.001		0.77	
	6 months	99	0.83 (4.4)	–3.24 (5.1)	4.1 (2.2-6.0)		<.001	<.001		0.86	
**ALT^c^, IU/L**											
	3 months	70	–18.7 (26.7)	–37.2 (36.2)	18.5 (3.3-33.8)		.02	.02		0.58	
	6 months	98	–10.0 (32.7)	–32.7 (38.4)	22.7 (8.8-36.6)		.002	.002		0.64	
**AST^d^, IU/L**											
	3 months	71	–9.18 (15.2)	–18.9 (23.8)	9.7 (0.2-19.3)		.046	.04		0.49	
	6 months	99	–6.35 (15.3)	–16.1 (24.5)	9.7 (1.6-17.9)		.02	.02		0.47	
**Systolic blood pressure (mmHg)**											
	3 months	61	1.1 (12.6)	–13.4 (15.2)	14.5 (7.4-21.6)		<.001	<.001		1.04	
	6 months	70	–2.4 (12.4)	–11.8 (15.0)	9.4 (2.9-16.0)		.005	.01		0.68	
**Diastolic blood pressure (mmHg)**											
	3 months	61	2.3 (7.7)	–6.1 (10.5)	8.4 (3.8-12.9)		.001	<.001		0.91	
	6 months	70	–0.9 (10.0)	–6.5 (9.0)	7.4 (2.8-11.9)		.002	.006		0.78	

^a^Adjusted for age, gender, and ethnicity.

^b^BMI: body mass index.

^e^ALT: alanine aminotransferase.

^d^AST: aspartate aminotransferase.

### Waist Circumference

Participants in the intervention group had greater reductions in waist circumference compared to those in the control group at 3 months. These greater reductions in waist circumference remained statistically significant at 6 months in both analyses, with a large effect size observed at 6 months in the per-protocol analysis (Table 4).

### Liver Enzymes

Both ALT and AST levels decreased markedly at 3 and 6 months in both the intention-to-treat and per-protocol analyses, irrespective of treatment group. However, there were significantly greater reductions of ALT and AST in the intervention group compared to the control group at 6 months, which remained after further adjustments for age, gender, and ethnicity in both analyses. Between-group differences in AST showed a small effect size (Cohen *d*<0.5), whereas a moderate effect size in ALT was observed at 6 months in both the intention-to-treat and per-protocol analyses (Tables 3 and 4).

### Blood Pressure

In both the intention-to-treat and per-protocol analyses, participants with hypertension in the intervention group had significantly greater reductions in systolic and diastolic blood pressure from baseline at 3 and 6 months as compared to those receiving standard care. After including all enrolled participants and adjusting for covariates, those assigned to the mobile app program were able to reduce their systolic and diastolic blood pressure to a markedly greater extent than those receiving standard care by an average of 15 mmHg and 9 mmHg, respectively, at 3 months. Using the same analysis methods, this difference remained statistically significant at 6 months. The effect size for both systolic and diastolic pressure was large at 3 months (Cohen *d*>0.8 for all) and was moderate for systolic blood pressure at 6 months (Tables 3 and 4).

### Mobile App Utilization

Data on app use of the 49 participants in the intervention group were obtained from the app developer. One account was deleted by the participant upon completing the program and was unavailable due to the privacy protection feature of the app. Overall, we observed a high percentage of active users in the intervention, with 76% (37/49) of participants showing a daily log-in of 137 days over a 182-day period (>75.3% of the total time). The average log-in days for the first 3 months, 4-6 months, and overall were 79.6 days (SD 17.9), 71.1 days (SD 25.6), and 151 days (SD 41.1), respectively. The mean percentage of log-in days was 87.6% (SD 19.6) in the first 3 months and decreased to 78.1% (SD 28.2) at 4-6 months. Furthermore, meal and weight logging were at 56.7% (SD 51.6) and 77.0% (SD 28.5) of the recommended utilization rate of daily and twice a week, respectively.

## Discussion

### Principal Findings

This study demonstrated that a 6-month mobile-enabled lifestyle intervention was able to produce clinically meaningful outcomes in patients with NAFLD. Patients enrolled in the 6-month nBuddy mobile app intervention program had a 5-fold higher likelihood of achieving ≥5% weight loss as compared to those receiving standard care. In addition, the mobile-enabled lifestyle intervention appeared to have a positive influence on components of surrogate markers of NAFLD such as waist circumference and BMI, along with improvements in liver enzymes (AST, ALT) and blood pressure. These positive results remained significant after an intention-to-treat approach, suggesting a notable effect among NAFLD patients. Our findings support the consensus that a modest weight loss of about 5% of baseline body weight within a 6-month period is associated with clinically meaningful reductions in liver enzymes [11,13,34].

Weight loss, along with control of metabolic risk factors, is the cornerstone of management of NAFLD in the absence of effective medical therapy. Current practice guidelines advocate a target weight loss of 7%-10% to achieve improvement in steatosis and inflammation [11,35]. In a Cuban study, a weight loss of ≥5% led to a resolution of steatohepatitis and reduction in the NAFLD activity score of at least 2 points, without worsening of fibrosis; however, regression of fibrosis occurred in those with ≥10% weight loss [36]. Hence, the greater the extent of weight loss with lifestyle changes, the more significant the improvement in NASH and fibrosis.

The use of mobile apps and online platforms as part of weight management in patients with noncommunicable diseases is increasingly growing in popularity. This stems partly from its ability to overcome issues faced by conventional weight management programs that require physical attendance. Health care providers observed a high nonattendance rate for patients with NAFLD requiring dietetics intervention and follow up [37,38]. Jiandani et al [39] reported a high attrition rate for in-house weight management programs whereby 34% of the participants discontinued after a single visit. The reasons cited included limited ability to pay for program services, limited time, and transportation challenges in attending appointments with health care professionals [40]. Other barriers included the lack of motivation and both patient and physician time constraints. In this study, the attrition rate of the intervention group using the mobile app (5%) was markedly lower than that of conventional face-to-face dietetics intervention cited in the literature [39]. This novel approach is also effective in addressing health behavior changes, thus leading to weight loss and beneficial outcomes [41]. The use of mobile apps similarly benefits health care practitioners through facilitating relationships with patients by enhancing the speed and ease of communication [42]. Use of a mobile app further provides practitioners with additional information in a timely manner that can improve patient care [42].

Previous studies have demonstrated the effectiveness of weight loss programs targeted at NAFLD patients in achieving a ≥5%-10% weight loss with a normalization of liver enzymes [43,44]. However, their scalability to a larger population poses a challenge. The use of mobile apps offers an alternative that might be scalable, more logistically friendly, and cost-effective with comparable or better results [45]. In fact, a recent meta-analysis on weight loss interventions in adults with chronic diseases found a markedly greater weight reduction of 2.5 kg among users in the mobile app group as compared with those in the nonmobile app group [45]. Another study examining lifestyle changes in patients with NAFLD reported an average weight reduction of 3.4 kg from baseline weight at 6 months via an online remote coaching method [34]. Similarly, our results demonstrated a mean weight loss of 4.1 kg over the 6-month period in participants who completed the mobile app program, which corresponded to a mean 5.1% reduction in weight. Our findings suggest that a mobile app with multifaceted automated algorithms and remote coaching is capable of helping patients with NAFLD lose a significant amount of weight.

Decreased aminotransferase levels have consistently been associated with histological improvement in NAFLD and are used as surrogate markers of inflammation. A study involving intensive nutrition counseling on patients with NAFLD found an average reduction of 33.1 IU/L and 15.4 IU/L in ALT and AST, respectively, at 12 months [15]. Although the current study did not have sufficient power to evaluate liver enzymes, our results suggest a similar trend, whereby participants enrolled in the mobile app intervention group had a mean reduction of 33.5 IU/L and 17.4 IU/L in ALT and AST, respectively, at 6 months. These improvements were also significantly greater when compared to those of the control group. In addition, Straznicky et al [46] found a concomitant 20% reduction in ALT along with 8% weight loss in participants receiving dietary intervention at 3 months [46]. Our results suggest a 2-fold (44%) reduction of ALT compared to the previous study, at approximately half (4%) the weight loss achieved at 3 and 6 months. This observation could be contributed by the differences in ethnicities between the two cohorts considering the higher visceral adiposity at a lower BMI in the Asian population [47,48]. This also suggests that Asian patients with NAFLD may benefit more from weight loss, with an augmented response to reduction in liver enzymes. The effectiveness of a mobile app–enabled lifestyle intervention can also consequently be seen as comparable to conventional weight loss programs.

Our study showed a significant difference in reduction of waist circumference (3.6 cm) in intervention as compared to control participants at 6 months using an intention-to-treat analysis. Pooled analysis of existing literature showed a greater mean reduction in waist circumference of 2.5 cm among mobile app users [45]. Our results also concur with another study showing an average greater reduction of 3.9 cm among users in a mobile phone weight loss program as compared to those in the control group at 12 months [49].

It is well-established that weight loss is correlated to reductions in blood pressure. A meta-analysis of randomized controlled trials on the influence of weight changes on blood pressure found an average of 1 mmHg reduction in systolic and diastolic blood pressure per kilogram of weight loss [50]. In a recent pilot study, systolic and diastolic blood pressure among mobile app users with a mean of 9.4% weight loss were 6 mmHg and 4 mmHg, respectively, at 3 months [49]. Our results corroborate with these findings and substantiate the benefits of blood pressure control in patients with NAFLD involved in a mobile app–enabled weight loss program.

### Strengths and Limitations

This study has several strengths. The use of randomized stratification ensured that the baseline characteristics of the study participants were similar between the control and intervention groups. Use of an adjusted intention-to-treat analysis model provided a more realistic indicator of the potential for program success when extrapolated to a clinical setting. This study is one of the first randomized controlled trials to investigate the effects of a mobile app utilizing a spectrum of evidence-based strategies in facilitating weight loss and improvement of relevant physiological and biochemical markers among patients with NAFLD. This intervention employed a mobile app that includes local dishes in the database, as well as culturally specific healthier alternatives. Having been developed with professional input, it fills the gap for research-tested apps developed with health care professional involvement, particularly in an Asian context [21].

This modality of intervention delivered through a well-designed mobile app was successful in achieving significant weight loss in NAFLD patients. Therefore, findings from this study can guide the successful incorporation of a mobile app into conventional lifestyle interventions for patients with NAFLD. Notably, the use of a mobile app in the NAFLD dietetics clinic has augmented service delivery in NUH since its inception. This program also has the potential to be applied in the management of other chronic diseases such as diabetes and cardiovascular diseases, as well as being extended to a wider population. Furthermore, our data suggest that a 3-month nBuddy app liver restoration program has the potential of yielding clinically meaningful weight loss in patients with NAFLD should a shorter time frame be desired.

The study also has a few limitations. First, this was a single-center trial with an intervention that precluded nonsmartphone users and those illiterate in English. Although smartphone ownership is rising, it continues to be dependent on education attainment and household income [51]. This trial might have thus included a smaller proportion of people of lower socioeconomic status. Along with the exclusion of nonEnglish users, this restricts the generalizability of findings to the greater population. Owing to the lack of research-tested culturally specific mobile apps for nonEnglish users, it would be worthwhile to explore different language versions of the mobile app should they be made available [52].

Second, the intervention continued to require the expertise of dietitians in coaching participants via the dashboard. However, the enablement of one dietitian in supporting multiple patients remotely may render the intervention more cost-effective as compared to face-to-face consultations. There is potential to scale up the solution considering the automated features such as provision of alternative healthier food options in the app, which can facilitate behavior change without the costs associated with intensive face-to-face counseling. Moreover, the delivery of advice by different health care practitioners in both groups also limits the ability to attribute the results solely to the mobile app. Nevertheless, this trial offers insight into the feasibility of introducing a combined dietitian and mobile app model in outpatient care settings where dietitians may not yet have a clear role. Finally, it would have been preferable for all patients enrolled in the study to have biopsy-proven NASH and an end-of-treatment biopsy. However, this was limited by funding and time constraints.

### Conclusions

Lifestyle intervention enabled by a mobile app can be effective in improving weight loss and anthropometric and clinical indices in patients with NAFLD. This study provides new insights on the feasibility and effectiveness of mobile apps in facilitating lifestyle interventions in NAFLD, along with further guidance for the application of this treatment modality to other chronic diseases. Health care professionals seeking to provide weight loss interventions to patients with NAFLD can now consider employing the use of a mobile app in effecting behavioral changes, including self-monitoring. Future studies can investigate the effectiveness of this treatment modality on a wider population, as well as evaluate important histological outcomes such as liver fibrosis.

## Appendix

Multimedia Appendix 1 CONSORT-EHEALTH V 1.6.1 checklist.

## Abbreviations

ALT: alanine aminotransferase
AST: aspartate aminotransferase
BMI: body mass index
CONSORT: Consolidated Standards of Reporting Trials
NAFLD: non-alcoholic fatty liver disease
NASH: non-alcoholic steatohepatitis
nBuddy: Nutritionist Buddy
NUH: National University Hospital
WHO: World Health Organization
