# Supplementary material for: Lifestyle Intervention Enabled by Mobile Technology on Weight Loss in Patients With Nonalcoholic Fatty Liver Disease: Randomized Controlled Trial
Source: JMIR Mhealth Uhealth. 2020 Apr 13;8(4):e14802. doi: 10.2196/14802 (PMC7186867; doi:10.2196/14802)
Supplement: Multimedia Appendix 1 [file mhealth_v8i4e14802_app1.pdf]

CONSORT-EHEALTH (V 1.6.1) - Submission/Publication Form

The CONSORT-EHEALTH checklist is intended for authors of randomized trials evaluating web-based and Internet-based applications/interventions, including mobile interventions, electronic games (incl multiplayer games), social media, certain telehealth applications, and other interactive and/or networked electronic applications. Some of the items (e.g. all subitems under item 5 - description of the intervention) may also be applicable for other study designs.

The goal of the CONSORT EHEALTH checklist and guideline is to be
a) a guide for reporting for authors of RCTs,
b) to form a basis for appraisal of an ehealth trial (in terms of validity)

CONSORT-EHEALTH items/subitems are MANDATORY reporting items for studies published in the Journal of Medical Internet Research and other journals / scientific societies endorsing the checklist.

Items numbered 1., 2., 3., 4a., 4b etc are original CONSORT or CONSORT-NPT (non-pharmacologic treatment) items.
Items with Roman numerals (i., ii, iii, iv etc.) are CONSORT-EHEALTH extensions/clarifications.

As the CONSORT-EHEALTH checklist is still considered in a formative stage, we would ask that you also RATE ON A SCALE OF 1-5 how important/useful you feel each item is FOR THE PURPOSE OF THE CHECKLIST and reporting guideline (optional).

Mandatory reporting items are marked with a red *.
In the textboxes, either copy & paste the relevant sections from your manuscript into this form - please include any quotes from your manuscript in QUOTATION MARKS,
or answer directly by providing additional information not in the manuscript, or elaborating on why the item was not relevant for this study.

YOUR ANSWERS WILL BE PUBLISHED AS A SUPPLEMENTARY FILE TO YOUR PUBLICATION IN JMIR AND ARE CONSIDERED PART OF YOUR PUBLICATION (IF ACCEPTED).
Please fill in these questions diligently. Information will not be copyedited, so please use proper spelling and grammar, use correct capitalization, and avoid abbreviations.

DO NOT FORGET TO SAVE AS PDF _AND_ CLICK THE SUBMIT BUTTON SO YOUR ANSWERS ARE IN OUR DATABASE !!!

Citation Suggestion (if you append the pdf as Appendix we suggest to cite this paper in the caption):
Eysenbach G, CONSORT-EHEALTH Group
CONSORT-EHEALTH: Improving and Standardizing Evaluation Reports of Web-based and Mobile Health Interventions
J Med Internet Res 2011;13(4):e126
URL: http://www.jmir.org/2011/4/e126/

doi: 10.2196/jmir.1923
PMID: 22209829

* Required

Your name *

First Last

Su Lin Lim

Primary Affiliation (short), City, Country *

University of Toronto, Toronto, Canada

National University Hospital, Singapore

Your e-mail address *

abc@gmail.com

su_lin_lim@nuhs.edu.sg

Title of your manuscript *

Provide the (draft) title of your manuscript.

Lifestyle Intervention enabled by Mobile Technology on Weight Loss in Patients with Non-alcoholic Fatty Liver Disease: Randomized Controlled Trial

Name of your App/Software/Intervention *

If there is a short and a long/alternate name, write the short name first and add the long name in brackets.

nBuddy (Nutritionist Buddy)

Evaluated Version (if any)

e.g. "V1", "Release 2017-03-01", "Version 2.0.27913"

Version 3.1.10.349

Language(s) *

What language is the intervention/app in? If multiple languages are available, separate by comma (e.g. "English, French")

English

URL of your Intervention Website or App

e.g. a direct link to the mobile app on app in appstore (itunes, Google Play), or URL of the website. If the intervention is a DVD or hardware, you can also link to an Amazon page.

https://apps.apple.com/sg/app/nutritionist-bu

URL of an image/screenshot (optional)

https://apps.apple.com/sg/app/nutritionist-bu

Accessibility *

Can an enduser access the intervention presently?

- ○ access is free and open
- ○ access only for special usergroups, not open
- ◉ access is open to everyone, but requires payment/subscription/in-app purchases
- ○ app/intervention no longer accessible
- ○ Other:

Primary Medical Indication/Disease/Condition *

e.g. "Stress", "Diabetes", or define the target group in brackets after the condition, e.g. "Autism (Parents of children with)", "Alzheimers (Informal Caregivers of)"

Non-alcoholic Fatty Liver Disease

Primary Outcomes measured in trial *

comma-separated list of primary outcomes reported in the trial

Weight loss

Secondary/other outcomes

Are there any other outcomes the intervention is expected to affect?

BMI, blood pressure, waist circumference, and liver enzymes

Recommended "Dose" *

What do the instructions for users say on how often the app should be used?

- (selected) Approximately Daily
- Approximately Weekly
- Approximately Monthly
- Approximately Yearly
- "as needed"
- Other:

Approx. Percentage of Users (starters) still using the app as recommended after 3 months *

- (selected) unknown / not evaluated
- 0-10%
- 11-20%
- 21-30%
- 31-40%
- 41-50%
- 51-60%
- 61-70%
- 71%-80%
- 81-90%
- 91-100%
- Other:

Overall, was the app/intervention effective? *

- (selected) yes: all primary outcomes were significantly better in intervention group vs control
- partly: SOME primary outcomes were significantly better in intervention group vs control
- no statistically significant difference between control and intervention
- potentially harmful: control was significantly better than intervention in one or more outcomes
- inconclusive: more research is needed
- Other:

Article Preparation Status/Stage *

At which stage in your article preparation are you currently (at the time you fill in this form)

- ○ not submitted yet - in early draft status
- ○ not submitted yet - in late draft status, just before submission
- ○ submitted to a journal but not reviewed yet
- ◉ submitted to a journal and after receiving initial reviewer comments
- ○ submitted to a journal and accepted, but not published yet
- ○ published
- ○ Other:

Journal *

If you already know where you will submit this paper (or if it is already submitted), please provide the journal name (if it is not JMIR, provide the journal name under "other")

- ○ not submitted yet / unclear where I will submit this
- ◉ Journal of Medical Internet Research (JMIR)
- ○ JMIR mHealth and UHealth
- ○ JMIR Serious Games
- ○ JMIR Mental Health
- ○ JMIR Public Health
- ○ JMIR Formative Research
- ○ Other JMIR sister journal
- ○ Other:

Is this a full powered effectiveness trial or a pilot/feasibility trial? *

- ◯ Pilot/feasibility
- ◉ Fully powered

Manuscript tracking number *

If this is a JMIR submission, please provide the manuscript tracking number under "other" (The ms tracking number can be found in the submission acknowledgement email, or when you login as author in JMIR. If the paper is already published in JMIR, then the ms tracking number is the four-digit number at the end of the DOI, to be found at the bottom of each published article in JMIR)

- ◯ no ms number (yet) / not (yet) submitted to / published in JMIR
- ◉ Other: 14802

TITLE AND ABSTRACT

1a) TITLE: Identification as a randomized trial in the title

1a) Does your paper address CONSORT item 1a? *

I.e does the title contain the phrase "Randomized Controlled Trial"? (if not, explain the reason under "other")

- ◉ yes
- ◯ Other:

1a-i) Identify the mode of delivery in the title

Identify the mode of delivery. Preferably use “web-based” and/or “mobile” and/or “electronic game” in the title. Avoid ambiguous terms like “online”, “virtual”, “interactive”. Use “Internet-based” only if Intervention includes non-web-based Internet components (e.g. email), use “computer-based” or “electronic” only if offline products are used. Use “virtual” only in the context of “virtual reality” (3-D worlds). Use “online” only in the context of “online support groups”. Complement or substitute product names with broader terms for the class of products (such as “mobile” or “smart phone” instead of “iphone”), especially if the application runs on different platforms.

	1	2	3	4	5	
subitem not at all important	○	○	○	○	◉	essential

Does your paper address subitem 1a-i? *

Copy and paste relevant sections from manuscript title (include quotes in quotation marks "like this" to indicate direct quotes from your manuscript), or elaborate on this item by providing additional information not in the ms, or briefly explain why the item is not applicable/relevant for your study

Yes, "mobile".

1a-ii) Non-web-based components or important co-interventions in title

Mention non-web-based components or important co-interventions in title, if any (e.g., “with telephone support”).

	1	2	3	4	5	
subitem not at all important	○	○	○	○	◉	essential

Does your paper address subitem 1a-ii?

Copy and paste relevant sections from manuscript title (include quotes in quotation marks "like this" to indicate direct quotes from your manuscript), or elaborate on this item by providing additional information not in the ms, or briefly explain why the item is not applicable/relevant for your study

Yes, "mobile technology".

1a-iii) Primary condition or target group in the title

Mention primary condition or target group in the title, if any (e.g., “for children with Type I Diabetes”) Example: A Web-based and Mobile Intervention with Telephone Support for Children with Type I Diabetes: Randomized Controlled Trial

	1	2	3	4	5	
subitem not at all important	○	○	○	○	◉	essential

Does your paper address subitem 1a-iii? *

Copy and paste relevant sections from manuscript title (include quotes in quotation marks "like this" to indicate direct quotes from your manuscript), or elaborate on this item by providing additional information not in the ms, or briefly explain why the item is not applicable/relevant for your study

Yes, "non-alcoholic fatty liver disease".

1b) ABSTRACT: Structured summary of trial design, methods, results, and conclusions

NPT extension: Description of experimental treatment, comparator, care providers, centers, and blinding status.

1b-i) Key features/functionalities/components of the intervention and comparator in the METHODS section of the ABSTRACT

Mention key features/functionalities/components of the intervention and comparator in the abstract. If possible, also mention theories and principles used for designing the site. Keep in mind the needs of systematic reviewers and indexers by including important synonyms. (Note: Only report in the abstract what the main paper is reporting. If this information is missing from the main body of text, consider adding it)

	1	2	3	4	5	
subitem not at all important	○	○	○	○	◉	essential

Does your paper address subitem 1b-i? *

Copy and paste relevant sections from the manuscript abstract (include quotes in quotation marks "like this" to indicate direct quotes from your manuscript), or elaborate on this item by providing additional information not in the ms, or briefly explain why the item is not applicable/relevant for your study

Yes, participants were "randomly allocated to either a control group (n=53) receiving standard care consisting of dietary and lifestyle advice by a trained nurse, or an intervention group (n=55) utilising Nutritionist Buddy (nBuddy) mobile app in addition to receiving dietary and lifestyle advice by a dietitian".

1b-ii) Level of human involvement in the METHODS section of the ABSTRACT

Clarify the level of human involvement in the abstract, e.g., use phrases like "fully automated" vs. "therapist/nurse/care provider/physician-assisted" (mention number and expertise of providers involved, if any). (Note: Only report in the abstract what the main paper is reporting. If this information is missing from the main body of text, consider adding it)

	1	2	3	4	5	
subitem not at all important	○	○	●	○	○	essential

Does your paper address subitem 1b-ii?

Copy and paste relevant sections from the manuscript abstract (include quotes in quotation marks "like this" to indicate direct quotes from your manuscript), or elaborate on this item by providing additional information not in the ms, or briefly explain why the item is not applicable/relevant for your study

Yes, participants were "randomly allocated to either a control group (n=53) receiving standard care consisting of dietary and lifestyle advice by a trained nurse, or an intervention group (n=55) utilising Nutritionist Buddy (nBuddy) mobile app in addition to receiving dietary and lifestyle advice by a dietitian".

1b-iii) Open vs. closed, web-based (self-assessment) vs. face-to-face assessments in the METHODS section of the ABSTRACT

Mention how participants were recruited (online vs. offline), e.g., from an open access website or from a clinic or a closed online user group (closed usergroup trial), and clarify if this was a purely web-based trial, or there were face-to-face components (as part of the intervention or for assessment). Clearly say if outcomes were self-assessed through questionnaires (as common in web-based trials). Note: In traditional offline trials, an open trial (open-label trial) is a type of clinical trial in which both the researchers and participants know which treatment is being administered. To avoid confusion, use “blinded” or “unblinded” to indicated the level of blinding instead of “open”, as “open” in web-based trials usually refers to “open access” (i.e. participants can self-enrol). (Note: Only report in the abstract what the main paper is reporting. If this information is missing from the main body of text, consider adding it)

	1	2	3	4	5	
subitem not at all important	○	○	○	◉	○	essential

Does your paper address subitem 1b-iii?

Copy and paste relevant sections from the manuscript abstract (include quotes in quotation marks "like this" to indicate direct quotes from your manuscript), or elaborate on this item by providing additional information not in the ms, or briefly explain why the item is not applicable/relevant for your study

Yes, participants were recruited "from a fatty liver outpatient clinic" and "randomly allocated to either a control group (n=53) receiving standard care consisting of dietary and lifestyle advice by a trained nurse, or an intervention group (n=55) utilising Nutritionist Buddy (nBuddy) mobile app in addition to receiving dietary and lifestyle advice by a dietitian".

1b-iv) RESULTS section in abstract must contain use data

Report number of participants enrolled/assessed in each group, the use/uptake of the intervention (e.g., attrition/adherence metrics, use over time, number of logins etc.), in addition to primary/secondary outcomes. (Note: Only report in the abstract what the main paper is reporting. If this information is missing from the main body of text, consider adding it)

	1	2	3	4	5	
subitem not at all important	○	○	○	○	◉	essential

Does your paper address subitem 1b-iv?

Copy and paste relevant sections from the manuscript abstract (include quotes in quotation marks "like this" to indicate direct quotes from your manuscript), or elaborate on this item by providing additional information not in the ms, or briefly explain why the item is not applicable/relevant for your study

Yes, this is addressed earlier in the methods section of the abstract, with “108 adults with NAFLD” allocated to either “control group (n=53)” or “intervention group (n=55)”.

1b-v) CONCLUSIONS/DISCUSSION in abstract for negative trials

Conclusions/Discussions in abstract for negative trials: Discuss the primary outcome - if the trial is negative (primary outcome not changed), and the intervention was not used, discuss whether negative results are attributable to lack of uptake and discuss reasons. (Note: Only report in the abstract what the main paper is reporting. If this information is missing from the main body of text, consider adding it)

	1	2	3	4	5	
subitem not at all important	(●)	( )	( )	( )	( )	essential

Does your paper address subitem 1b-v?

Copy and paste relevant sections from the manuscript abstract (include quotes in quotation marks "like this" to indicate direct quotes from your manuscript), or elaborate on this item by providing additional information not in the ms, or briefly explain why the item is not applicable/relevant for your study

It is not a negative trial.

INTRODUCTION

2a) In INTRODUCTION: Scientific background and explanation of rationale

2a-i) Problem and the type of system/solution

Describe the problem and the type of system/solution that is object of the study: intended as stand-alone intervention vs. incorporated in broader health care program? Intended for a particular patient population? Goals of the intervention, e.g., being more cost-effective to other interventions, replace or complement other solutions? (Note: Details about the intervention are provided in “Methods” under 5)

	1	2	3	4	5	
subitem not at all important	○	○	○	○	◉	essential

Does your paper address subitem 2a-i? *

Copy and paste relevant sections from the manuscript (include quotes in quotation marks "like this" to indicate direct quotes from your manuscript), or elaborate on this item by providing additional information not in the ms, or briefly explain why the item is not applicable/relevant for your study

Yes,

“In Asia, an estimated 20%-30% of the adult population have NAFLD, with a higher prevalence among obese patients. The increase in prevalence can be attributed to a shift in dietary and lifestyle habits brought about by rapid globalisation.”

And:

“By mitigating the barriers associated with committing to repeated in-house nutrition and exercise therapy sessions, mobile app increases the potential of reach and efficacy of lifestyle interventions.”

And:

“The objectives of our study were thus to evaluate the effect of lifestyle intervention consisting of dietary and physical activity modifications enabled by mobile app in facilitating weight loss and improving relevant health indicators in patients with NAFLD.”

2a-ii) Scientific background, rationale: What is known about the (type of) system

Scientific background, rationale: What is known about the (type of) system that is the object of the study (be sure to discuss the use of similar systems for other conditions/diagnoses, if appropiate), motivation for the study, i.e. what are the reasons for and what is the context for this specific study, from which stakeholder viewpoint is the study performed, potential impact of findings [2]. Briefly justify the choice of the comparator.

	1	2	3	4	5	
subitem not at all important	○	○	○	◉	○	essential

Does your paper address subitem 2a-ii? *

Copy and paste relevant sections from the manuscript (include quotes in quotation marks "like this" to indicate direct quotes from your manuscript), or elaborate on this item by providing additional information not in the ms, or briefly explain why the item is not applicable/relevant for your study

Yes, it is acknowledged that the success of face-to-face weight loss interventions is "dependent on the intensity of nutrition counselling and frequency of visits to dietitians and exercise therapists", which "renders the treatment modality resource-intensive and costly, with high attrition rates, limited reach and scalability".

2b) In INTRODUCTION: Specific objectives or hypotheses

Does your paper address CONSORT subitem 2b? *

Copy and paste relevant sections from the manuscript (include quotes in quotation marks "like this" to indicate direct quotes from your manuscript), or elaborate on this item by providing additional information not in the ms, or briefly explain why the item is not applicable/relevant for your study

Yes. "The objectives of our study were thus to evaluate the effect of lifestyle intervention consisting of dietary and physical activity modifications enabled by mobile app in facilitating weight loss and improving relevant health indicators in patients with NAFLD."

METHODS

3a) Description of trial design (such as parallel, factorial) including allocation ratio

Does your paper address CONSORT subitem 3a? *

Copy and paste relevant sections from the manuscript (include quotes in quotation marks "like this" to indicate direct quotes from your manuscript), or elaborate on this item by providing additional information not in the ms, or briefly explain why the item is not applicable/relevant for your study

Yes. It is a "parallel randomized controlled trial" with an "allocation ratio of 1:1".

3b) Important changes to methods after trial commencement (such as eligibility criteria), with reasons

Does your paper address CONSORT subitem 3b? *

Copy and paste relevant sections from the manuscript (include quotes in quotation marks "like this" to indicate direct quotes from your manuscript), or elaborate on this item by providing additional information not in the ms, or briefly explain why the item is not applicable/relevant for your study

No, as no changes to methods were made after trial commencement.

3b-i) Bug fixes, Downtimes, Content Changes

Bug fixes, Downtimes, Content Changes: ehealth systems are often dynamic systems. A description of changes to methods therefore also includes important changes made on the intervention or comparator during the trial (e.g., major bug fixes or changes in the functionality or content) (5-iii) and other “unexpected events” that may have influenced study design such as staff changes, system failures/downtimes, etc. [2].

	1	2	3	4	5	
subitem not at all important	○	○	●	○	○	essential

Does your paper address subitem 3b-i?

Copy and paste relevant sections from the manuscript (include quotes in quotation marks "like this" to indicate direct quotes from your manuscript), or elaborate on this item by providing additional information not in the ms, or briefly explain why the item is not applicable/relevant for your study

There were no major downtimes or bug fixes during the period of the trial.

4a) Eligibility criteria for participants

Does your paper address CONSORT subitem 4a? *

Copy and paste relevant sections from the manuscript (include quotes in quotation marks "like this" to indicate direct quotes from your manuscript), or elaborate on this item by providing additional information not in the ms, or briefly explain why the item is not applicable/relevant for your study

Yes.

"Adults above 21 years of age who had been diagnosed with NAFLD confirmed by the presence of steatosis in the liver on ultrasound, with Body Mass Index (BMI) more than or equivalent to 23 kg/m2, able to read and write in English, and who owned a smartphone with data plan, were included in the study. Exclusion criteria were consumption of more than one and a half times the limit of alcohol recommended for the population (alcohol dose 15 g/day for women and 30 g/day for men) and those infected with hepatitis B or C virus. Patients who were pregnant, receiving hepatotoxic medication, with cirrhosis, poorly-controlled diabetes mellitus (HbA1c >10%), diabetes needing insulin, recent cardiovascular event in the past 6 months, stage 4 and above kidney disease, concomitant liver disease, depression, untreated hypothyroidism, heart failure and clinically or biochemically recognized systemic diseases, were also excluded from the study."

4a-i) Computer / Internet literacy

Computer / Internet literacy is often an implicit “de facto” eligibility criterion - this should be explicitly clarified.

	1	2	3	4	5	
subitem not at all important	○	○	○	○	◉	essential

Does your paper address subitem 4a-i?

Copy and paste relevant sections from the manuscript (include quotes in quotation marks "like this" to indicate direct quotes from your manuscript), or elaborate on this item by providing additional information not in the ms, or briefly explain why the item is not applicable/relevant for your study

Yes, only participants who were "able to read and write in English, and who owned a smartphone with data plan, were included in the study".

4a-ii) Open vs. closed, web-based vs. face-to-face assessments:

Open vs. closed, web-based vs. face-to-face assessments: Mention how participants were recruited (online vs. offline), e.g., from an open access website or from a clinic, and clarify if this was a purely web-based trial, or there were face-to-face components (as part of the intervention or for assessment), i.e., to what degree got the study team to know the participant. In online-only trials, clarify if participants were quasi-anonymous and whether having multiple identities was possible or whether technical or logistical measures (e.g., cookies, email confirmation, phone calls) were used to detect/prevent these.

	1	2	3	4	5	
subitem not at all important	○	○	○	○	●	essential

Does your paper address subitem 4a-ii? *

Copy and paste relevant sections from the manuscript (include quotes in quotation marks "like this" to indicate direct quotes from your manuscript), or elaborate on this item by providing additional information not in the ms, or briefly explain why the item is not applicable/relevant for your study

Yes, "patients were recruited from an NAFLD clinic in NUH through referrals from clinicians after screening", with “participants allocated to intervention group were each provided with advice on dietary and lifestyle modification by a dietitian via a single face-to-face session in clinic followed by remote support through a mobile app for a 6-month period” while “participants randomized to the control group were provided with usual standard care, which consisted of advice on diet and lifestyle modification by a nurse trained in diet counselling via a single face-to-face session in the NAFLD clinic”.

4a-iii) Information giving during recruitment

Information given during recruitment. Specify how participants were briefed for recruitment and in the informed consent procedures (e.g., publish the informed consent documentation as appendix, see also item X26), as this information may have an effect on user self-selection, user expectation and may also bias results.

	1	2	3	4	5	
subitem not at all important	○	○	○	○	●	essential

Does your paper address subitem 4a-iii?

Copy and paste relevant sections from the manuscript (include quotes in quotation marks "like this" to indicate direct quotes from your manuscript), or elaborate on this item by providing additional information not in the ms, or briefly explain why the item is not applicable/relevant for your study

Yes. "A written informed consent was obtained from each patient before enrolment."

4b) Settings and locations where the data were collected

Does your paper address CONSORT subitem 4b? *

Copy and paste relevant sections from the manuscript (include quotes in quotation marks "like this" to indicate direct quotes from your manuscript), or elaborate on this item by providing additional information not in the ms, or briefly explain why the item is not applicable/relevant for your study

Yes.

"This parallel randomized controlled trial was conducted between July 2017 and November 2018 at National University Hospital (NUH), a tertiary university hospital in Singapore. Patients were recruited from an NAFLD clinic in NUH through referrals from clinicians after screening."

And:

"All outcomes were part of routine measurements taken by trained nurses and blood tests conducted at the outpatient NAFLD clinic."

4b-i) Report if outcomes were (self-)assessed through online questionnaires

Clearly report if outcomes were (self-)assessed through online questionnaires (as common in web-based trials) or otherwise.

	1	2	3	4	5	
subitem not at all important	○	○	◉	○	○	essential

Does your paper address subitem 4b-i? *

Copy and paste relevant sections from the manuscript (include quotes in quotation marks "like this" to indicate direct quotes from your manuscript), or elaborate on this item by providing additional information not in the ms, or briefly explain why the item is not applicable/relevant for your study

Not applicable, as outcomes were assessed from visits to an outpatient clinic.

4b-ii) Report how institutional affiliations are displayed

Report how institutional affiliations are displayed to potential participants [on ehealth media], as affiliations with prestigious hospitals or universities may affect volunteer rates, use, and reactions with regards to an intervention.(Not a required item – describe only if this may bias results)

	1	2	3	4	5	
subitem not at all important	○	○	●	○	○	essential

Does your paper address subitem 4b-ii?

Copy and paste relevant sections from the manuscript (include quotes in quotation marks "like this" to indicate direct quotes from your manuscript), or elaborate on this item by providing additional information not in the ms, or briefly explain why the item is not applicable/relevant for your study

Recruitment was not carried out through public or media channels. The mobile application used in our intervention cohort does not bear the name of the hospital or hospital's organisation.

5) The interventions for each group with sufficient details to allow replication, including how and when they were actually administered

5-i) Mention names, credential, affiliations of the developers, sponsors, and owners

Mention names, credential, affiliations of the developers, sponsors, and owners [6] (if authors/evaluators are owners or developer of the software, this needs to be declared in a “Conflict of interest” section or mentioned elsewhere in the manuscript).

	1	2	3	4	5	
subitem not at all important	○	○	○	○	●	essential

Does your paper address subitem 5-i?

Copy and paste relevant sections from the manuscript (include quotes in quotation marks "like this" to indicate direct quotes from your manuscript), or elaborate on this item by providing additional information not in the ms, or briefly explain why the item is not applicable/relevant for your study

Yes. "The app was conceptualized by the principal investigator and developed by Verita Analytics."

5-ii) Describe the history/development process

Describe the history/development process of the application and previous formative evaluations (e.g., focus groups, usability testing), as these will have an impact on adoption/use rates and help with interpreting results.

	1	2	3	4	5	
subitem not at all important	○	○	●	○	○	essential

Does your paper address subitem 5-ii?

Copy and paste relevant sections from the manuscript (include quotes in quotation marks "like this" to indicate direct quotes from your manuscript), or elaborate on this item by providing additional information not in the ms, or briefly explain why the item is not applicable/relevant for your study

Yes.

"The app was conceptualized by the principal investigator and developed by Verita Analytics. It was developed using the Obesity-Related Behavioural Intervention Trials (ORBIT) Model for behavioral treatment as a framework for translating behavioral science discoveries into treatments as it is a flexible and robust process, to design, conduct and evaluate mobile-app based behavioral interventions."

5-iii) Revisions and updating

Revisions and updating. Clearly mention the date and/or version number of the application/intervention (and comparator, if applicable) evaluated, or describe whether the intervention underwent major changes during the evaluation process, or whether the development and/or content was “frozen” during the trial. Describe dynamic components such as news feeds or changing content which may have an impact on the replicability of the intervention (for unexpected events see item 3b).

	1	2	3	4	5	
subitem not at all important	○	○	●	○	○	essential

Does your paper address subitem 5-iii?

Copy and paste relevant sections from the manuscript (include quotes in quotation marks "like this" to indicate direct quotes from your manuscript), or elaborate on this item by providing additional information not in the ms, or briefly explain why the item is not applicable/relevant for your study

The version number was not included in the manuscript, as changes to the app since inception and over the course of the study have been minor so far.

5-iv) Quality assurance methods

Provide information on quality assurance methods to ensure accuracy and quality of information provided [1], if applicable.

	1	2	3	4	5	
subitem not at all important	○	○	●	○	○	essential

Does your paper address subitem 5-iv?

Copy and paste relevant sections from the manuscript (include quotes in quotation marks "like this" to indicate direct quotes from your manuscript), or elaborate on this item by providing additional information not in the ms, or briefly explain why the item is not applicable/relevant for your study

Yes, participants in the intervention group “were each provided with advice on dietary and lifestyle modification by a dietitian via a single face-to-face session in clinic followed by remote support through a mobile app for a 6-month period” and “taught to utilize the Nutritionist Buddy (nBuddy)—a mobile app to track diet and physical activity and induce behavioral changes to achieve optimal weight”, with the app “developed using the Obesity-Related Behavioural Intervention Trials (ORBIT) Model for behavioral treatment as a framework”.

Meanwhile, participants in control group were “provided with usual standard care, which consisted of advice on diet and lifestyle modification by a nurse trained in diet counselling via a single face-to-face session in the NAFLD clinic, with a syllabus similar to that provided in the face-to-face visit with the dietitian in the intervention group”.

5-v) Ensure replicability by publishing the source code, and/or providing screenshots/screen-capture video, and/or providing flowcharts of the algorithms used

Ensure replicability by publishing the source code, and/or providing screenshots/screen-capture video, and/or providing flowcharts of the algorithms used. Replicability (i.e., other researchers should in principle be able to replicate the study) is a hallmark of scientific reporting.

	1	2	3	4	5	
subitem not at all important	○	○	○	●	○	essential

Does your paper address subitem 5-v?

Copy and paste relevant sections from the manuscript (include quotes in quotation marks "like this" to indicate direct quotes from your manuscript), or elaborate on this item by providing additional information not in the ms, or briefly explain why the item is not applicable/relevant for your study

Yes, screenshots are provided in Figure 1, with information on the mobile app employed in the intervention, "nutritionist Buddy (nBuddy)—a mobile application (app) created by Verita Analytics”. The app functions utilised are also described in detail in the manuscript as follow:
“• A food diary logging system, coupled with individualized caloric goals based on users’ age, gender and physical activity level, allows self-monitoring of intake.
• The auto-recording of daily steps via syncing with users’ mobile devices’ in-built pedometer enables self-monitoring of physical activity. Step goal increases automatically each week, from initial 3000 to 10,000 steps by the third week of usage. A range of physical activities can be logged in manually if exercises were done in the absence of mobile devices.
• A weight logging function encourages self-tracking of weight loss progression.
• A dashboard enables dietitians to monitor users’ input (ie, food intake and physical activity) and progress (ie, weight) to provide real-time feedback and encouragement.
• A peer support chat channel allows users to connect with selected family members and peers to bolster user motivation.
• A video viewing function delivers weekly educational clips.
• An automated response system evaluates suitability of food choices and provides instantaneous feedback, generating a list of healthier and culturally appropriate alternatives via an algorithm.
• Daily, weekly, and monthly graph reports on weight, calorie intake and steps facilitate the tracking of progress.
• Scripted daily tips and timed automated reminders prompt users to log in daily meal intake and weekly weight.”

5-vi) Digital preservation

Digital preservation: Provide the URL of the application, but as the intervention is likely to change or disappear over the course of the years; also make sure the intervention is archived (Internet Archive, webcitation.org, and/or publishing the source code or screenshots/videos alongside the article). As pages behind login screens cannot be archived, consider creating demo pages which are accessible without login.

	1	2	3	4	5	
subitem not at all important	○	○	○	○	◉	essential

Does your paper address subitem 5-vi?

Copy and paste relevant sections from the manuscript (include quotes in quotation marks "like this" to indicate direct quotes from your manuscript), or elaborate on this item by providing additional information not in the ms, or briefly explain why the item is not applicable/relevant for your study

Yes, https://apps.apple.com/sg/app/nutritionist-buddy/id1144069608.

5-vii) Access

Access: Describe how participants accessed the application, in what setting/context, if they had to pay (or were paid) or not, whether they had to be a member of specific group. If known, describe how participants obtained “access to the platform and Internet” [1]. To ensure access for editors/reviewers/readers, consider to provide a “backdoor” login account or demo mode for reviewers/readers to explore the application (also important for archiving purposes, see vi).

	1	2	3	4	5	
subitem not at all important	○	○	○	●	○	essential

Does your paper address subitem 5-vii? *

Copy and paste relevant sections from the manuscript (include quotes in quotation marks "like this" to indicate direct quotes from your manuscript), or elaborate on this item by providing additional information not in the ms, or briefly explain why the item is not applicable/relevant for your study

Yes. "The app is available commercially in the app stores, with basic features accessible for free. Payment is required to unlock additional features such as videos, daily tips and nutritionist support. Full features of the app were made available to the intervention participants as part of collaboration with Verita Analytics."

5-viii) Mode of delivery, features/functionalities/components of the intervention and comparator, and the theoretical framework

Describe mode of delivery, features/functionalities/components of the intervention and comparator, and the theoretical framework [6] used to design them (instructional strategy [1], behaviour change techniques, persuasive features, etc., see e.g., [7, 8] for terminology). This includes an in-depth description of the content (including where it is coming from and who developed it) [1]," whether [and how] it is tailored to individual circumstances and allows users to track their progress and receive feedback" [6]. This also includes a description of communication delivery channels and – if computer-mediated communication is a component – whether communication was synchronous or asynchronous [6]. It also includes information on presentation strategies [1], including page design principles, average amount of text on pages, presence of hyperlinks to other resources, etc. [1].

	1	2	3	4	5	
subitem not at all important	○	○	○	◉	○	essential

Does your paper address subitem 5-viii? *

Copy and paste relevant sections from the manuscript (include quotes in quotation marks "like this" to indicate direct quotes from your manuscript), or elaborate on this item by providing additional information not in the ms, or briefly explain why the item is not applicable/relevant for your study

Yes.

“Participants allocated to intervention group were each provided with advice on dietary and lifestyle modification by a dietitian via a single face-to-face session in clinic followed by remote support through a mobile app for a 6-month period. They were taught to utilize the Nutritionist Buddy (nBuddy)—a mobile app to track diet and physical activity and induce behavioral changes to achieve optimal weight. The app was conceptualized by the principal investigator and developed by Verita Analytics. It was developed using the Obesity-Related Behavioural Intervention Trials (ORBIT) Model for behavioral treatment as a framework for translating behavioral science discoveries into treatments as it is a flexible and robust process, to design, conduct and evaluate mobile-app based behavioral interventions.”

And:

“The set of in-built functions in nBuddy as described below is an amalgamation of evidence-based behavioral modification strategies to promote weight loss/maintenance:

- A food diary logging system, coupled with individualized caloric goals based on users' age, gender and physical activity level, allows self-monitoring of intake.
- The auto-recording of daily steps via syncing with users' mobile devices' in-built pedometer enables self-monitoring of physical activity. Step goal increases automatically each week, from initial 3000 to 10,000 steps by the third week of usage. A range of physical activities can be logged in manually if exercises were done in the absence of mobile devices.
- A weight logging function encourages self-tracking of weight loss progression.
- A dashboard enables dietitians to monitor users' input (ie, food intake and physical activity) and progress (ie, weight) to provide real-time feedback and encouragement.
- A peer support chat channel allows users to connect with selected family members and peers to bolster user motivation.
- A video viewing function delivers weekly educational clips.
- An automated response system evaluates suitability of food choices and provides instantaneous feedback, generating a list of healthier and culturally appropriate alternatives via an algorithm.
- Daily, weekly, and monthly graph reports on weight, calorie intake and steps facilitate the tracking of progress.
- Scripted daily tips and timed automated reminders prompt users to log in daily meal intake and weekly weight.”

And:

“Participants randomized to the control group were provided with usual standard care, which consisted of advice on diet and lifestyle modification by a nurse trained in diet counselling via a single face-to-face session in the NAFLD clinic, with a syllabus similar to that provided in the face-to-face visit with the dietitian in the intervention group.”

5-ix) Describe use parameters

Describe use parameters (e.g., intended “doses” and optimal timing for use). Clarify what instructions or recommendations were given to the user, e.g., regarding timing, frequency, heaviness of use, if any, or was the intervention used ad libitum.

	1	2	3	4	5	
subitem not at all important	○	○	○	◉	○	essential

Does your paper address subitem 5-ix?

Copy and paste relevant sections from the manuscript (include quotes in quotation marks "like this" to indicate direct quotes from your manuscript), or elaborate on this item by providing additional information not in the ms, or briefly explain why the item is not applicable/relevant for your study

Yes, “participants allocated to intervention group were each provided with advice on dietary and lifestyle modification by a dietitian via a single face-to-face session in clinic followed by remote support through a mobile app for a 6-month period. They were taught to utilize the Nutritionist Buddy (nBuddy)—a mobile app to track diet and physical activity and induce behavioral changes to achieve optimal weight”, with “timed automated reminders prompt users to log in daily meal intake and weekly weight”, as well as step goal which “increases automatically each week, from initial 3000 to 10,000 steps by the third week of usage”.

5-x) Clarify the level of human involvement

Clarify the level of human involvement (care providers or health professionals, also technical assistance) in the e-intervention or as co-intervention (detail number and expertise of professionals involved, if any, as well as “type of assistance offered, the timing and frequency of the support, how it is initiated, and the medium by which the assistance is delivered”. It may be necessary to distinguish between the level of human involvement required for the trial, and the level of human involvement required for a routine application outside of a RCT setting (discuss under item 21 – generalizability).

	1	2	3	4	5	
subitem not at all important	○	○	○	◉	○	essential

Does your paper address subitem 5-x?

Copy and paste relevant sections from the manuscript (include quotes in quotation marks "like this" to indicate direct quotes from your manuscript), or elaborate on this item by providing additional information not in the ms, or briefly explain why the item is not applicable/relevant for your study

Yes. Participants in the intervention group were provided "remote support through a mobile app for a 6-month period" by dietitians, where "a dashboard enables dietitians to monitor users' input (ie, food intake and physical activity) and progress (ie, weight) to provide real-time feedback and encouragement."

5-xi) Report any prompts/reminders used

Report any prompts/reminders used: Clarify if there were prompts (letters, emails, phone calls, SMS) to use the application, what triggered them, frequency etc. It may be necessary to distinguish between the level of prompts/reminders required for the trial, and the level of prompts/reminders for a routine application outside of a RCT setting (discuss under item 21 – generalizability).

	1	2	3	4	5	
subitem not at all important	○	○	○	◉	○	essential

Does your paper address subitem 5-xi? *

Copy and paste relevant sections from the manuscript (include quotes in quotation marks "like this" to indicate direct quotes from your manuscript), or elaborate on this item by providing additional information not in the ms, or briefly explain why the item is not applicable/relevant for your study

Yes, there were "timed automated reminders prompt users to log in daily meal intake and weekly weight", as part of the app features.

5-xii) Describe any co-interventions (incl. training/support)

Describe any co-interventions (incl. training/support): Clearly state any interventions that are provided in addition to the targeted eHealth intervention, as ehealth intervention may not be designed as stand-alone intervention. This includes training sessions and support [1]. It may be necessary to distinguish between the level of training required for the trial, and the level of training for a routine application outside of a RCT setting (discuss under item 21 – generalizability.

	1	2	3	4	5	
subitem not at all important	○	○	○	◉	○	essential

Does your paper address subitem 5-xii? *

Copy and paste relevant sections from the manuscript (include quotes in quotation marks "like this" to indicate direct quotes from your manuscript), or elaborate on this item by providing additional information not in the ms, or briefly explain why the item is not applicable/relevant for your study

Yes, in addition to the mobile application use, "participants allocated to intervention group were each provided with advice on dietary and lifestyle modification by a dietitian via a single face-to-face session in clinic" at the initial visit, which is similar in syllabus to that provided to NAFLD clinic patients in the "usual standard care, which consisted of advice on diet and lifestyle modification by a nurse trained in diet counselling".

6a) Completely defined pre-specified primary and secondary outcome measures, including how and when they were assessed

Does your paper address CONSORT subitem 6a? *

Copy and paste relevant sections from the manuscript (include quotes in quotation marks "like this" to indicate direct quotes from your manuscript), or elaborate on this item by providing additional information not in the ms, or briefly explain why the item is not applicable/relevant for your study

Yes. "All outcomes were part of routine measurements taken by trained nurses and blood tests conducted at the outpatient NAFLD clinic. Assessors were not blinded to the groups allocated for the study participants. Body weight was measured using a calibrated digital weighing machine (Seca 767, Germany) to the nearest 0.1 kilogram. Height was measured in meters to two decimal points using the stadiometer which was attached to the Seca scale and the corresponding BMI was calculated. Waist circumference was measured using a tape measure at the midpoint between the lower margin of the last palpable rib and the top of the iliac crest as recommended by the World Health Organization [30]. Alanine aminotransferase (ALT) and aspartate aminotransferase (AST) were determined by an automated kinetic method. Blood pressure (for participants with hypertension) was measured using a standard mercury sphygmomanometer. Participants' characteristics such as age, gender, ethnicity and existing relevant co-morbidities were also collected at baseline."

6a-i) Online questionnaires: describe if they were validated for online use and apply CHERRIES items to describe how the questionnaires were designed/deployed

If outcomes were obtained through online questionnaires, describe if they were validated for online use and apply CHERRIES items to describe how the questionnaires were designed/deployed [9].

	1	2	3	4	5	
subitem not at all important	○	○	◉	○	○	essential

Does your paper address subitem 6a-i?

Copy and paste relevant sections from manuscript text

No online questionnaires were used.

6a-ii) Describe whether and how “use” (including intensity of use/dosage) was defined/measured/monitored

Describe whether and how “use” (including intensity of use/dosage) was defined/measured/monitored (logins, logfile analysis, etc.). Use/adoption metrics are important process outcomes that should be reported in any ehealth trial.

	1	2	3	4	5	
subitem not at all important	○	○	○	◉	○	essential

Does your paper address subitem 6a-ii?

Copy and paste relevant sections from manuscript text

Yes.

"Data of 49 users in the intervention group were obtained from the app developer."

And:

"Overall, we observed a high percentage of active users in the intervention, with 76% (37/49) of participants with daily log-in of 137 days over a 182-day period (>75.3% of the time). The average log-in days for the first 3 months, 4-6 months and overall were 79.6 days (SD 17.9), 71.1 days (SD 25.6) and 151 days (SD 41.1), respectively. The mean percentage of log-in days was 87.6% (SD 19.6) in the first 3 months and decreased to 78.1% (SD 28.2) at 4-6 months. Furthermore, meal and weight logging were at 56.7% (SD 51.6) and 77.0% (SD 28.5) of the recommended utilisation rate of daily and twice a week respectively."

6a-iii) Describe whether, how, and when qualitative feedback from participants was obtained

Describe whether, how, and when qualitative feedback from participants was obtained (e.g., through emails, feedback forms, interviews, focus groups).

	1	2	3	4	5	
subitem not at all important	○	○	●	○	○	essential

Does your paper address subitem 6a-iii?

Copy and paste relevant sections from manuscript text

Qualitative feedback was not collected.

6b) Any changes to trial outcomes after the trial commenced, with reasons

Does your paper address CONSORT subitem 6b? *

Copy and paste relevant sections from the manuscript (include quotes in quotation marks "like this" to indicate direct quotes from your manuscript), or elaborate on this item by providing additional information not in the ms, or briefly explain why the item is not applicable/relevant for your study

There were no changes to trial outcomes after the trial commenced.

7a) How sample size was determined

NPT: When applicable, details of whether and how the clustering by care provides or centers was addressed

7a-i) Describe whether and how expected attrition was taken into account when calculating the sample size

Describe whether and how expected attrition was taken into account when calculating the sample size.

	1	2	3	4	5	
subitem not at all important	○	○	○	○	◉	essential

Does your paper address subitem 7a-i?

Copy and paste relevant sections from manuscript title (include quotes in quotation marks "like this" to indicate direct quotes from your manuscript), or elaborate on this item by providing additional information not in the ms, or briefly explain why the item is not applicable/relevant for your study

Yes. "Studies that provided nutrition therapy interventions targeted at weight loss in patients with NAFLD were referenced for sample size calculation. The primary unit of interest is at least a 5% weight loss by 6-month. It was postulated that 10% of the control subjects will achieve this successful outcome and the intervention would increase this by 4-fold. With 90% power at 5% significance level, and allowing for a 10% dropout rate, a sample size of 100 (50 per group) would be required."

7b) When applicable, explanation of any interim analyses and stopping guidelines

Does your paper address CONSORT subitem 7b? *

Copy and paste relevant sections from the manuscript (include quotes in quotation marks "like this" to indicate direct quotes from your manuscript), or elaborate on this item by providing additional information not in the ms, or briefly explain why the item is not applicable/relevant for your study

No interim analysis was done. Participants were recruited on a voluntary basis and may exit the study anytime. No adverse events were recorded during the period of the study as well.

8a) Method used to generate the random allocation sequence

NPT: When applicable, how care providers were allocated to each trial group

Does your paper address CONSORT subitem 8a? *

Copy and paste relevant sections from the manuscript (include quotes in quotation marks "like this" to indicate direct quotes from your manuscript), or elaborate on this item by providing additional information not in the ms, or briefly explain why the item is not applicable/relevant for your study

Yes. "Stratified randomization of screened participants was carried out, with participants first stratified by gender, age (<40 years old or ≥40 years old) and BMI (<27.5 kg/m2 or ≥27.5 kg/m2). Within each stratum, participants were assigned to either control or intervention group based on drawing from sealed opaque envelopes with allocation ratio of 1:1, prepared by a third party not involved in the study and blinded to the study objectives according to the Consolidated Standards of Reporting Trials (CONSORT) statement."

8b) Type of randomisation; details of any restriction (such as blocking and block size)

Does your paper address CONSORT subitem 8b? *

Copy and paste relevant sections from the manuscript (include quotes in quotation marks "like this" to indicate direct quotes from your manuscript), or elaborate on this item by providing additional information not in the ms, or briefly explain why the item is not applicable/relevant for your study

Yes. "Stratified randomization of screened participants was carried out, with participants first stratified by gender, age (<40 years old or ≥40 years old) and BMI (<27.5 kg/m2 or ≥27.5 kg/m2). Within each stratum, participants were assigned to either control or intervention group based on drawing from sealed opaque envelopes with allocation ratio of 1:1."

9) Mechanism used to implement the random allocation sequence (such as sequentially numbered containers), describing any steps taken to conceal the sequence until interventions were assigned

Does your paper address CONSORT subitem 9? *

Copy and paste relevant sections from the manuscript (include quotes in quotation marks "like this" to indicate direct quotes from your manuscript), or elaborate on this item by providing additional information not in the ms, or briefly explain why the item is not applicable/relevant for your study

Yes. "Within each stratum, participants were assigned to either control or intervention group based on drawing from sealed opaque envelopes with allocation ratio of 1:1, prepared by a third party not involved in the study and blinded to the study objectives according to the Consolidated Standards of Reporting Trials (CONSORT) statement."

10) Who generated the random allocation sequence, who enrolled participants, and who assigned participants to interventions

Does your paper address CONSORT subitem 10? *

Copy and paste relevant sections from the manuscript (include quotes in quotation marks "like this" to indicate direct quotes from your manuscript), or elaborate on this item by providing additional information not in the ms, or briefly explain why the item is not applicable/relevant for your study

Yes. Participants were enrolled by members of the research team. They were "assigned to either control or intervention group based on drawing from sealed opaque envelopes with allocation ratio of 1:1, prepared by a third party not involved in the study and blinded to the study objectives according to the Consolidated Standards of Reporting Trials (CONSORT) statement".

11a) If done, who was blinded after assignment to interventions (for example, participants, care providers, those assessing outcomes) and how

NPT: Whether or not administering co-interventions were blinded to group assignment

11a-i) Specify who was blinded, and who wasn't

Specify who was blinded, and who wasn't. Usually, in web-based trials it is not possible to blind the participants [1, 3] (this should be clearly acknowledged), but it may be possible to blind outcome assessors, those doing data analysis or those administering co-interventions (if any).

	1	2	3	4	5	
subitem not at all important	○	○	○	◉	○	essential

Does your paper address subitem 11a-i? *

Copy and paste relevant sections from the manuscript (include quotes in quotation marks "like this" to indicate direct quotes from your manuscript), or elaborate on this item by providing additional information not in the ms, or briefly explain why the item is not applicable/relevant for your study

Participants were not blinded to study objectives as it was necessary to brief them about the time commitment should they be randomised into the mobile application group. Research dietitians and nurses were not blinded as well because of the need to deliver interventions after participants were being randomised.
Meanwhile, subsequent referrals to the dietitian by the physicians and outpatient dietetic follow-up visits outside of the NAFLD clinic were not affected by participation in this study, as these providers were not involved in this study and by default, blinded to its objectives

11a-ii) Discuss e.g., whether participants knew which intervention was the "intervention of interest" and which one was the "comparator"

Informed consent procedures (4a-ii) can create biases and certain expectations - discuss e.g., whether participants knew which intervention was the "intervention of interest" and which one was the "comparator".

	1	2	3	4	5	
subitem not at all important	○	○	○	◉	○	essential

Does your paper address subitem 11a-ii?

Copy and paste relevant sections from the manuscript (include quotes in quotation marks "like this" to indicate direct quotes from your manuscript), or elaborate on this item by providing additional information not in the ms, or briefly explain why the item is not applicable/relevant for your study

The comparator is the part of the standard care provided at the outpatient fatty liver clinic throughout the period of the study. The participants could not be blinded to which is the comparator or intervention of interest as the standard care would have been made known to them when they saw their physician as part of the routine medical management by the physicians.

11b) If relevant, description of the similarity of interventions

(this item is usually not relevant for ehealth trials as it refers to similarity of a placebo or sham intervention to a active medication/intervention)

Does your paper address CONSORT subitem 11b? *

Copy and paste relevant sections from the manuscript (include quotes in quotation marks "like this" to indicate direct quotes from your manuscript), or elaborate on this item by providing additional information not in the ms, or briefly explain why the item is not applicable/relevant for your study

Yes.

"To evaluate the effect of lifestyle intervention enabled by mobile app in facilitating weight loss, participants allocated to intervention group were each provided with advice on dietary and lifestyle modification by a dietitian via a single face-to-face session in clinic followed by remote support through a mobile app for a 6-month period."

And:

"Participants randomized to the control group were provided with usual standard care, which consisted of advice on diet and lifestyle modification by a nurse trained in diet counselling via a single face-to-face session in the NAFLD clinic, with a syllabus similar to that provided in the face-to-face visit with the dietitian in the intervention group."

12a) Statistical methods used to compare groups for primary and secondary outcomes

NPT: When applicable, details of whether and how the clustering by care providers or centers was addressed

Does your paper address CONSORT subitem 12a? *

Copy and paste relevant sections from the manuscript (include quotes in quotation marks "like this" to indicate direct quotes from your manuscript), or elaborate on this item by providing additional information not in the ms, or briefly explain why the item is not applicable/relevant for your study

Yes.

"Results were expressed as mean and standard deviation for normally distributed variables, and median and interquartile range for variables that did not satisfy normality criteria. Categorical data were expressed as frequencies and percentages. To compare baseline characteristics, and 3- and 6-month changes between groups, chi-square and independent samples t test were used for categorical and continuous variables respectively. Between-group differences in the numerical and binary outcomes were compared using General Linear Model and Poisson Regression Model respectively, adjusting for age, gender and ethnicity. Between-group Cohen d effect sizes were calculated with (Mpost – Mpre)/SDpooled."

12a-i) Imputation techniques to deal with attrition / missing values

Imputation techniques to deal with attrition / missing values: Not all participants will use the intervention/comparator as intended and attrition is typically high in ehealth trials. Specify how participants who did not use the application or dropped out from the trial were treated in the statistical analysis (a complete case analysis is strongly discouraged, and simple imputation techniques such as LOCF may also be problematic [4]).

	1	2	3	4	5	
subitem not at all important	○	○	●	○	○	essential

Does your paper address subitem 12a-i? *

Copy and paste relevant sections from the manuscript (include quotes in quotation marks "like this" to indicate direct quotes from your manuscript), or elaborate on this item by providing additional information not in the ms, or briefly explain why the item is not applicable/relevant for your study

No imputation was carried out. "Per-protocol analysis was carried out on 101 patients who completed the study, while intention-to-treat analysis was carried out among all 108 patients assigned to the original groups, where data was available for each of the parameters."

12b) Methods for additional analyses, such as subgroup analyses and adjusted analyses

Does your paper address CONSORT subitem 12b? *

Copy and paste relevant sections from the manuscript (include quotes in quotation marks "like this" to indicate direct quotes from your manuscript), or elaborate on this item by providing additional information not in the ms, or briefly explain why the item is not applicable/relevant for your study

Yes. "Between-group differences in the numerical and binary outcomes were compared using General Linear Model and Poisson Regression Model respectively, adjusting for age, gender and ethnicity."

X26) REB/IRB Approval and Ethical Considerations [recommended as subheading under "Methods"] (not a CONSORT item)

X26-i) Comment on ethics committee approval

	1	2	3	4	5	
subitem not at all important	○	○	○	○	◉	essential

Does your paper address subitem X26-i?

Copy and paste relevant sections from the manuscript (include quotes in quotation marks "like this" to indicate direct quotes from your manuscript), or elaborate on this item by providing additional information not in the ms, or briefly explain why the item is not applicable/relevant for your study

Yes. "This study was conducted in accordance with the Declaration of Helsinki, and received ethical approval from the National Healthcare Group Domain Specific Review Board in Singapore. It was registered at ANZCTR.org.au, number ACTRN12617001001381."

x26-ii) Outline informed consent procedures

Outline informed consent procedures e.g., if consent was obtained offline or online (how? Checkbox, etc.?), and what information was provided (see 4a-ii). See [6] for some items to be included in informed consent documents.

	1	2	3	4	5	
subitem not at all important	○	○	○	○	◉	essential

Does your paper address subitem X26-ii?

Copy and paste relevant sections from the manuscript (include quotes in quotation marks "like this" to indicate direct quotes from your manuscript), or elaborate on this item by providing additional information not in the ms, or briefly explain why the item is not applicable/relevant for your study

Yes. "A written informed consent was obtained from each patient before enrolment."

X26-iii) Safety and security procedures

Safety and security procedures, incl. privacy considerations, and any steps taken to reduce the likelihood or detection of harm (e.g., education and training, availability of a hotline)

	1	2	3	4	5	
subitem not at all important	○	○	◉	○	○	essential

Does your paper address subitem X26-iii?

Copy and paste relevant sections from the manuscript (include quotes in quotation marks "like this" to indicate direct quotes from your manuscript), or elaborate on this item by providing additional information not in the ms, or briefly explain why the item is not applicable/relevant for your study

To address privacy considerations, intervention participants were encouraged to use nicknames instead of real names when they signed up for accounts on the mobile app.

RESULTS

13a) For each group, the numbers of participants who were randomly assigned, received intended treatment, and were analysed for the primary outcome

NPT: The number of care providers or centers performing the intervention in each group and the number of patients treated by each care provider in each center

Does your paper address CONSORT subitem 13a? *

Copy and paste relevant sections from the manuscript (include quotes in quotation marks "like this" to indicate direct quotes from your manuscript), or elaborate on this item by providing additional information not in the ms, or briefly explain why the item is not applicable/relevant for your study

Yes.

"A total of 154 subjects referred were screened for participation. Forty-six did not meet the eligibility criteria, primarily due to refusal to participate and lack of English literacy. The remaining 108 subjects were enrolled and randomized (55 intervention and 53 control participants). Seven of the enrolled participants withdrew from the study, of which 5 (4.6%) were from intervention and 2 (1.9%) from control. A total of 101 patients completed the study, with 50 allocated to the intervention group."

And:

"Per-protocol analysis was carried out on 101 patients who completed the study, while intention-to-treat analysis was carried out among all 108 patients assigned to the original groups, where data was available for each of the parameters."

13b) For each group, losses and exclusions after randomisation, together with reasons

Does your paper address CONSORT subitem 13b? (NOTE: Preferably, this is shown in a CONSORT flow diagram) *

Copy and paste relevant sections from the manuscript (include quotes in quotation marks "like this" to indicate direct quotes from your manuscript), or elaborate on this item by providing additional information not in the ms, or briefly explain why the item is not applicable/relevant for your study

Yes. "A total of 154 subjects referred were screened for participation. Forty-six did not meet the eligibility criteria, primarily due to refusal to participate and lack of English literacy. The remaining 108 subjects were enrolled and randomized (55 intervention and 53 control participants). Seven of the enrolled participants withdrew from the study, of which 5 (4.6%) were from intervention and 2 (1.9%) from control. A total of 101 patients completed the study, with 50 allocated to the intervention group." The reasons for exclusions are provided in Figure 2 CONSORT Flow Diagram.

13b-i) Attrition diagram

Strongly recommended: An attrition diagram (e.g., proportion of participants still logging in or using the intervention/comparator in each group plotted over time, similar to a survival curve) or other figures or tables demonstrating usage/dose/engagement.

	1	2	3	4	5	
subitem not at all important	○	○	○	◉	○	essential

Does your paper address subitem 13b-i?

Copy and paste relevant sections from the manuscript or cite the figure number if applicable (include quotes in quotation marks "like this" to indicate direct quotes from your manuscript), or elaborate on this item by providing additional information not in the ms, or briefly explain why the item is not applicable/relevant for your study

Yes. "Overall, we observed a high percentage of active users in the intervention, with 76% (37/49) of participants with daily log-in of 137 days over a 182-day period (>75.3% of the time). The average log-in days for the first 3 months, 4-6 months and overall were 79.6 days (SD 17.9), 71.1 days (SD 25.6) and 151 days (SD 41.1), respectively. The mean percentage of log-in days was 87.6% (SD 19.6) in the first 3 months and decreased to 78.1% (SD 28.2) at 4-6 months. Furthermore, meal and weight logging were at 56.7% (SD 51.6) and 77.0% (SD 28.5) of the recommended utilisation rate of daily and twice a week respectively."

14a) Dates defining the periods of recruitment and follow-up

Does your paper address CONSORT subitem 14a? *

Copy and paste relevant sections from the manuscript (include quotes in quotation marks "like this" to indicate direct quotes from your manuscript), or elaborate on this item by providing additional information not in the ms, or briefly explain why the item is not applicable/relevant for your study

Yes. "This parallel randomized controlled trial was conducted between July 2017 and November 2018 at National University Hospital (NUH), a tertiary university hospital in Singapore."

14a-i) Indicate if critical “secular events” fell into the study period

Indicate if critical “secular events” fell into the study period, e.g., significant changes in Internet resources available or “changes in computer hardware or Internet delivery resources”

	1	2	3	4	5	
subitem not at all important	(selected)					essential

Does your paper address subitem 14a-i?

Copy and paste relevant sections from the manuscript (include quotes in quotation marks "like this" to indicate direct quotes from your manuscript), or elaborate on this item by providing additional information not in the ms, or briefly explain why the item is not applicable/relevant for your study

There were no "secular events" that coincided with the study period, hence none was reported.

14b) Why the trial ended or was stopped (early)

Does your paper address CONSORT subitem 14b? *

Copy and paste relevant sections from the manuscript (include quotes in quotation marks "like this" to indicate direct quotes from your manuscript), or elaborate on this item by providing additional information not in the ms, or briefly explain why the item is not applicable/relevant for your study

The trial did not end earlier than planned.

15) A table showing baseline demographic and clinical characteristics for each group

NPT: When applicable, a description of care providers (case volume, qualification, expertise, etc.) and centers (volume) in each group

Does your paper address CONSORT subitem 15? *

Copy and paste relevant sections from the manuscript (include quotes in quotation marks "like this" to indicate direct quotes from your manuscript), or elaborate on this item by providing additional information not in the ms, or briefly explain why the item is not applicable/relevant for your study

Yes, "participants' baseline characteristics were summarised in Table 1".

15-i) Report demographics associated with digital divide issues

In ehealth trials it is particularly important to report demographics associated with digital divide issues, such as age, education, gender, social-economic status, computer/Internet/ehealth literacy of the participants, if known.

	1	2	3	4	5	
subitem not at all important	○	○	○	◉	○	essential

Does your paper address subitem 15-i? *

Copy and paste relevant sections from the manuscript (include quotes in quotation marks "like this" to indicate direct quotes from your manuscript), or elaborate on this item by providing additional information not in the ms, or briefly explain why the item is not applicable/relevant for your study

Yes, "participants' baseline characteristics were summarised in Table 1", including "age, gender and ethnicity".

16) For each group, number of participants (denominator) included in each analysis and whether the analysis was by original assigned groups

16-i) Report multiple "denominators" and provide definitions

Report multiple "denominators" and provide definitions: Report N's (and effect sizes) "across a range of study participation [and use] thresholds" [1], e.g., N exposed, N consented, N used more than x times, N used more than y weeks, N participants "used" the intervention/comparator at specific pre-defined time points of interest (in absolute and relative numbers per group). Always clearly define "use" of the intervention.

	1	2	3	4	5	
subitem not at all important	○	○	○	○	◉	essential

Does your paper address subitem 16-i? *

Copy and paste relevant sections from the manuscript (include quotes in quotation marks "like this" to indicate direct quotes from your manuscript), or elaborate on this item by providing additional information not in the ms, or briefly explain why the item is not applicable/relevant for your study

Yes.

"A total of 154 subjects referred were screened for participation. Forty-six did not meet the eligibility criteria, primarily due to refusal to participate and lack of English literacy. The remaining 108 subjects were enrolled and randomized (55 intervention and 53 control participants). Seven of the enrolled participants withdrew from the study, of which 5 (4.6%) were from intervention and 2 (1.9%) from control. A total of 101 patients completed the study, with 50 allocated to the intervention group."

And:

"Overall, we observed a high percentage of active users in the intervention, with 76% (37/49) of participants with daily log-in of 137 days over a 182-day period (>75.3% of the time). The average log-in days for the first 3 months, 4-6 months and overall were 79.6 days (SD 17.9), 71.1 days (SD 25.6) and 151 days (SD 41.1), respectively. The mean percentage of log-in days was 87.6% (SD 19.6) in the first 3 months and decreased to 78.1% (SD 28.2) at 4-6 months. Furthermore, meal and weight logging were at 56.7% (SD 51.6) and 77.0% (SD 28.5) of the recommended utilisation rate of daily and twice a week respectively."

16-ii) Primary analysis should be intent-to-treat

Primary analysis should be intent-to-treat, secondary analyses could include comparing only “users”, with the appropriate caveats that this is no longer a randomized sample (see 18-i).

	1	2	3	4	5	
subitem not at all important	○	○	○	○	◉	essential

Does your paper address subitem 16-ii?

Copy and paste relevant sections from the manuscript (include quotes in quotation marks "like this" to indicate direct quotes from your manuscript), or elaborate on this item by providing additional information not in the ms, or briefly explain why the item is not applicable/relevant for your study

Yes. "Per-protocol analysis was carried out on 101 patients who completed the study, while intention-to-treat analysis was carried out among all 108 patients assigned to the original groups, where data was available for each of the parameters."

Meanwhile, the abstract included only results from intention-to-treat analysis.

17a) For each primary and secondary outcome, results for each group, and the estimated effect size and its precision (such as 95% confidence interval)

Does your paper address CONSORT subitem 17a? *

Copy and paste relevant sections from the manuscript (include quotes in quotation marks "like this" to indicate direct quotes from your manuscript), or elaborate on this item by providing additional information not in the ms, or briefly explain why the item is not applicable/relevant for your study

The significance level, precision (e.g. 95% confidence interval), and estimated effect size of each of the primary and secondary outcomes were reported in table 3.

17a-i) Presentation of process outcomes such as metrics of use and intensity of use

In addition to primary/secondary (clinical) outcomes, the presentation of process outcomes such as metrics of use and intensity of use (dose, exposure) and their operational definitions is critical. This does not only refer to metrics of attrition (13-b) (often a binary variable), but also to more continuous exposure metrics such as “average session length”. These must be accompanied by a technical description how a metric like a “session” is defined (e.g., timeout after idle time) [1] (report under item 6a).

	1	2	3	4	5	
subitem not at all important	○	○	○	●	○	essential

Does your paper address subitem 17a-i?

Copy and paste relevant sections from the manuscript (include quotes in quotation marks "like this" to indicate direct quotes from your manuscript), or elaborate on this item by providing additional information not in the ms, or briefly explain why the item is not applicable/relevant for your study

Yes. "Overall, we observed a high percentage of active users in the intervention, with 76% (37/49) of participants with daily log-in of 137 days over a 182-day period (>75.3% of the time). The average log-in days for the first 3 months, 4-6 months and overall were 79.6 days (SD 17.9), 71.1 days (SD 25.6) and 151 days (SD 41.1), respectively. The mean percentage of log-in days was 87.6% (SD 19.6) in the first 3 months and decreased to 78.1% (SD 28.2) at 4-6 months. Furthermore, meal and weight logging were at 56.7% (SD 51.6) and 77.0% (SD 28.5) of the recommended utilisation rate of daily and twice a week respectively."

17b) For binary outcomes, presentation of both absolute and relative effect sizes is recommended

Does your paper address CONSORT subitem 17b? *

Copy and paste relevant sections from the manuscript (include quotes in quotation marks "like this" to indicate direct quotes from your manuscript), or elaborate on this item by providing additional information not in the ms, or briefly explain why the item is not applicable/relevant for your study

Yes. "Using intention-to-treat analysis, the intervention group had a 5-fold likelihood (RR: 5.2; P=.003; 95% CI:1.8-15.4) of achieving ≥5% weight loss when compared to control group at 6-month." Meanwhile, absolute effect size is presented in table 2.

18) Results of any other analyses performed, including subgroup analyses and adjusted analyses, distinguishing pre-specified from exploratory

Does your paper address CONSORT subitem 18? *

Copy and paste relevant sections from the manuscript (include quotes in quotation marks "like this" to indicate direct quotes from your manuscript), or elaborate on this item by providing additional information not in the ms, or briefly explain why the item is not applicable/relevant for your study

Yes, blood pressure of the hypertensive subgroup was analysed and presented. "In both intention-to-treat and per-protocol analyses, hypertensive participants in the intervention group had significantly greater reductions in systolic and diastolic blood pressure from baseline at 3- and 6-month, as compared to those receiving standard care. After including all enrolled participants and adjusting for covariates, those assigned to the mobile app program were able to reduce their systolic and diastolic blood pressure markedly greater than those receiving standard care by an average of 15 mmHg and 9 mmHg respectively at 3-month. Using the same analyses methods, the trend of observation remained statistically significant at 6-month. Effect size was large for both systolic and diastolic pressure at 3-month (Cohen d>0.8 for all), while moderate for systolic blood pressure at 6-month." Meanwhile, adjusted analyses were presented in tables 2 and 3.

18-i) Subgroup analysis of comparing only users

A subgroup analysis of comparing only users is not uncommon in ehealth trials, but if done, it must be stressed that this is a self-selected sample and no longer an unbiased sample from a randomized trial (see 16-iii).

	1	2	3	4	5	
subitem not at all important	○	○	◉	○	○	essential

Does your paper address subitem 18-i?

Copy and paste relevant sections from the manuscript (include quotes in quotation marks "like this" to indicate direct quotes from your manuscript), or elaborate on this item by providing additional information not in the ms, or briefly explain why the item is not applicable/relevant for your study

Both per-protocol and intention-to-treat analysis were done, which were distinguished clearly in the results. Meanwhile, no specific subgroup analysis was carried out to compare only users within the intervention with higher application usage.

19) All important harms or unintended effects in each group

(for specific guidance see CONSORT for harms)

Does your paper address CONSORT subitem 19? *

Copy and paste relevant sections from the manuscript (include quotes in quotation marks "like this" to indicate direct quotes from your manuscript), or elaborate on this item by providing additional information not in the ms, or briefly explain why the item is not applicable/relevant for your study

Not applicable, as there was no harm or adverse event reported during the study.

19-i) Include privacy breaches, technical problems

Include privacy breaches, technical problems. This does not only include physical “harm” to participants, but also incidents such as perceived or real privacy breaches [1], technical problems, and other unexpected/unintended incidents. “Unintended effects” also includes unintended positive effects [2].

	1	2	3	4	5	
subitem not at all important	○	○	○	◉	○	essential

Does your paper address subitem 19-i?

Copy and paste relevant sections from the manuscript (include quotes in quotation marks "like this" to indicate direct quotes from your manuscript), or elaborate on this item by providing additional information not in the ms, or briefly explain why the item is not applicable/relevant for your study

Not applicable. There was no event related to privacy breaches or major technical issue that occurred.

19-ii) Include qualitative feedback from participants or observations from staff/researchers

Include qualitative feedback from participants or observations from staff/researchers, if available, on strengths and shortcomings of the application, especially if they point to unintended/unexpected effects or uses. This includes (if available) reasons for why people did or did not use the application as intended by the developers.

	1	2	3	4	5	
subitem not at all important	○	○	◉	○	○	essential

Does your paper address subitem 19-ii?

Copy and paste relevant sections from the manuscript (include quotes in quotation marks "like this" to indicate direct quotes from your manuscript), or elaborate on this item by providing additional information not in the ms, or briefly explain why the item is not applicable/relevant for your study

Not applicable. There was no formal collection of qualitative feedback from participants or recording of observations.

DISCUSSION

22) Interpretation consistent with results, balancing benefits and harms, and considering other relevant evidence

NPT: In addition, take into account the choice of the comparator, lack of or partial blinding, and unequal expertise of care providers or centers in each group

22-i) Restate study questions and summarize the answers suggested by the data, starting with primary outcomes and process outcomes (use)

Restate study questions and summarize the answers suggested by the data, starting with primary outcomes and process outcomes (use).

	1	2	3	4	5	
subitem not at all important	○	○	○	○	◉	essential

Does your paper address subitem 22-i? *

Copy and paste relevant sections from the manuscript (include quotes in quotation marks "like this" to indicate direct quotes from your manuscript), or elaborate on this item by providing additional information not in the ms, or briefly explain why the item is not applicable/relevant for your study

Yes. "This study demonstrated that a 6-month mobile-enabled lifestyle intervention was able to produce clinically meaningful outcomes in NAFLD patients. We observed that NAFLD patients enrolled in the 6-month nBuddy mobile app program had a 5-fold likelihood of achieving ≥5% weight loss as compared to those receiving standard care. In addition, the mobile-enabled lifestyle intervention appeared to have a positive influence on components of surrogate markers of NAFLD, such as waist circumference and BMI, along with improvements in liver enzymes (AST, ALT) and blood pressure. The trend of these positive results remained after an intention-to-treat approach, suggesting a notable effect among NAFLD patients. Our study findings support the consensus that a modest weight loss of about 5% of baseline body weight within a period of 6-month is associated with clinically meaningful reductions in liver enzymes."

22-ii) Highlight unanswered new questions, suggest future research

Highlight unanswered new questions, suggest future research.

	1	2	3	4	5	
subitem not at all important	○	○	○	◉	○	essential

Does your paper address subitem 22-ii?

Copy and paste relevant sections from the manuscript (include quotes in quotation marks "like this" to indicate direct quotes from your manuscript), or elaborate on this item by providing additional information not in the ms, or briefly explain why the item is not applicable/relevant for your study

Yes. "Future studies can investigate the effectiveness of this treatment modality on a wider population, as well as evaluate important histological outcomes such as liver fibrosis."

20) Trial limitations, addressing sources of potential bias, imprecision, and, if relevant, multiplicity of analyses

20-i) Typical limitations in ehealth trials

Typical limitations in ehealth trials: Participants in ehealth trials are rarely blinded. Ehealth trials often look at a multiplicity of outcomes, increasing risk for a Type I error. Discuss biases due to non-use of the intervention/usability issues, biases through informed consent procedures, unexpected events.

	1	2	3	4	5	
subitem not at all important	○	○	○	◉	○	essential

Does your paper address subitem 20-i? *

Copy and paste relevant sections from the manuscript (include quotes in quotation marks "like this" to indicate direct quotes from your manuscript), or elaborate on this item by providing additional information not in the ms, or briefly explain why the item is not applicable/relevant for your study

Yes.

“The study has a few limitations. Firstly, it was a single centre trial with an intervention that precluded non-smartphone users and those illiterate in English. While smartphone ownership is rising, it continues to be dependent on education attainment and household income. The trial may thus include a lower proportion of lower socioeconomic status groups. Along with the exclusion of non-English users, this restricts the generalizability of findings to the greater population.”

And:

“Secondly, the intervention continued to require the expertise of dietitians in coaching participants via the dashboard.”

And:

“The delivery of advice by different healthcare practitioners in both groups also limits the ability to attribute results solely to the mobile app.”

And:

“Finally, the authors acknowledge that it would have been preferable for all patients enrolled in the study to have biopsy-proven NASH and a second biopsy done at the end of treatment. In this manner, study findings could have been more robust, by allowing the comparison of outcomes of NAFLD activity and fibrosis scores.”

21) Generalisability (external validity, applicability) of the trial findings

NPT: External validity of the trial findings according to the intervention, comparators, patients, and care providers or centers involved in the trial

21-i) Generalizability to other populations

Generalizability to other populations: In particular, discuss generalizability to a general Internet population, outside of a RCT setting, and general patient population, including applicability of the study results for other organizations

	1	2	3	4	5	
subitem not at all important	○	○	◉	○	○	essential

Does your paper address subitem 21-i?

Copy and paste relevant sections from the manuscript (include quotes in quotation marks "like this" to indicate direct quotes from your manuscript), or elaborate on this item by providing additional information not in the ms, or briefly explain why the item is not applicable/relevant for your study

Yes. "The study has a few limitations. Firstly, it was a single centre trial with an intervention that precluded non-smartphone users and those illiterate in English. While smartphone ownership is rising, it continues to be dependent on education attainment and household income. The trial may thus include a lower proportion of lower socioeconomic status groups. Along with the exclusion of non-English users, this restricts the generalizability of findings to the greater population."

21-ii) Discuss if there were elements in the RCT that would be different in a routine application setting

Discuss if there were elements in the RCT that would be different in a routine application setting (e.g., prompts/reminders, more human involvement, training sessions or other co-interventions) and what impact the omission of these elements could have on use, adoption, or outcomes if the intervention is applied outside of a RCT setting.

	1	2	3	4	5	
subitem not at all important	○	○	◉	○	○	essential

Does your paper address subitem 21-ii?

Copy and paste relevant sections from the manuscript (include quotes in quotation marks "like this" to indicate direct quotes from your manuscript), or elaborate on this item by providing additional information not in the ms, or briefly explain why the item is not applicable/relevant for your study

Yes, there were a few research dietitians supporting the participants through the dashboard in the RCT, which may not be possible in a routine application setting where manpower costs may be an issue.

OTHER INFORMATION

23) Registration number and name of trial registry

Does your paper address CONSORT subitem 23? *

Copy and paste relevant sections from the manuscript (include quotes in quotation marks "like this" to indicate direct quotes from your manuscript), or elaborate on this item by providing additional information not in the ms, or briefly explain why the item is not applicable/relevant for your study

Yes. "It was registered at ANZCTR.org.au, number ACTRN12617001001381".

24) Where the full trial protocol can be accessed, if available

Does your paper address CONSORT subitem 24? *

Cite a Multimedia Appendix, other reference, or copy and paste relevant sections from the manuscript (include quotes in quotation marks "like this" to indicate direct quotes from your manuscript), or elaborate on this item by providing additional information not in the ms, or briefly explain why the item is not applicable/relevant for your study

Yes, "https://www.anzctr.org.au/Trial/Registration/TrialReview.aspx?id=372937".

25) Sources of funding and other support (such as supply of drugs), role of funders

Does your paper address CONSORT subitem 25? *

Copy and paste relevant sections from the manuscript (include quotes in quotation marks "like this" to indicate direct quotes from your manuscript), or elaborate on this item by providing additional information not in the ms, or briefly explain why the item is not applicable/relevant for your study

Yes.

"The study was funded by National University Health System Allied Health and Nursing Grant and the National Medical Research Council Grant.
The authors would like to thank Dr Tan Poh Seng, Dr Ong Li Zhen, Dr Anita Lim, Dr Junita Lee, Ms Genevieve Yeo and nurses from the University Digestive Clinic, National University Hospital for supporting this study."

And:

"Full features of the app were made available to the intervention participants as part of collaboration with Verita Analytics."

X27) Conflicts of Interest (not a CONSORT item)

X27-i) State the relation of the study team towards the system being evaluated

In addition to the usual declaration of interests (financial or otherwise), also state the relation of the study team towards the system being evaluated, i.e., state if the authors/evaluators are distinct from or identical with the developers/sponsors of the intervention.

	1	2	3	4	5	
subitem not at all important	○	○	○	○	◉	essential

Does your paper address subitem X27-i?

Copy and paste relevant sections from the manuscript (include quotes in quotation marks "like this" to indicate direct quotes from your manuscript), or elaborate on this item by providing additional information not in the ms, or briefly explain why the item is not applicable/relevant for your study

Yes. "The app was conceptualized by the principal investigator and developed by Verita Analytics." The authors are distinct from the developers of the intervention.

About the CONSORT EHEALTH checklist

As a result of using this checklist, did you make changes in your manuscript? *

- ○ yes, major changes
- ◉ yes, minor changes
- ○ no

What were the most important changes you made as a result of using this checklist?

Changes in the explanation of application used in the trial.

How much time did you spend on going through the checklist INCLUDING making changes in your manuscript *

6 hours.

As a result of using this checklist, do you think your manuscript has improved? *

- ◉ yes
- ○ no
- ○ Other:

Would you like to become involved in the CONSORT EHEALTH group?

This would involve for example becoming involved in participating in a workshop and writing an "Explanation and Elaboration" document

- ○ yes
- ◉ no
- ○ Other:

Any other comments or questions on CONSORT EHEALTH

Nil.

STOP - Save this form as PDF before you click submit

To generate a record that you filled in this form, we recommend to generate a PDF of this page (on a Mac, simply select "print" and then select "print as PDF") before you submit it.

When you submit your (revised) paper to JMIR, please upload the PDF as supplementary file.

Don't worry if some text in the textboxes is cut off, as we still have the complete information in our database. Thank you!

Final step: Click submit !

Click submit so we have your answers in our database!

Submit

Never submit passwords through Google Forms.

This content is neither created nor endorsed by Google. Report Abuse - Terms of Service - Privacy Policy

Google Forms
